# IMPRESSION – prediction of NMR parameters for 3-dimensional chemical structures using machine learning with near quantum chemical accuracy†
†Electronic supplementary information (ESI) available. See DOI: 10.1039/c9sc03854j


**DOI:** 10.1039/c9sc03854j

**Published:** 2019-11-20

**Authors:** Will Gerrard, Lars A. Bratholm, Martin J. Packer, Adrian J. Mulholland, David R. Glowacki, Craig P. Butts

**Affiliations:** a University of Bristol , Bristol , UK . Email: craig.butts@bristol.ac.uk ; Email: glowacki@bristol.ac.uk; b Chemistry , R&D Oncology , AstraZeneca , Cambridge CB4 0QA , UK

## Abstract

The IMPRESSION (Intelligent Machine PREdiction of Shift and Scalar information Of Nuclei) machine learning system provides an efficient and accurate method for the prediction of NMR parameters from 3-dimensional molecular structures. Here we demonstrate that machine learning predictions of NMR parameters, trained on quantum chemical computed values, can be as accurate as, but computationally much more efficient (tens of milliseconds per molecular structure) than, quantum chemical calculations (hours/days per molecular structure) starting from the same 3-dimensional structure. Training the machine learning system on quantum chemical predictions, rather than experimental data, circumvents the need for the existence of large, structurally diverse, error-free experimental databases and makes IMPRESSION applicable to solving 3-dimensional problems such as molecular conformation and stereoisomerism.

## Abstract 2

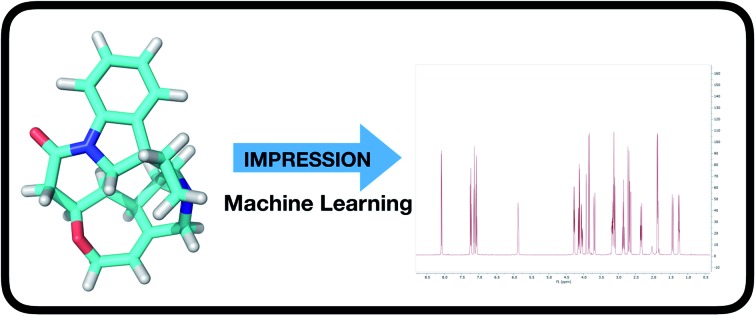
The IMPRESSION machine learning system can predict NMR parameters for 3D structures with similar results to DFT but in seconds rather than hours.

## 1 Introduction

NMR spectroscopy remains the pre-eminent analytical technique for elucidating molecular structure in solution, with the prediction and interpretation of ^1^H and ^13^C chemical shifts and scalar coupling constants playing a key role. The prediction of these parameters, especially in studies of 3-dimensional molecular structure, are increasingly moving towards quantitative comparison between computed values for proposed chemical structures and experiment. In such comparisons, the use of fast and accurate NMR prediction methods is crucial.

Fast empirical predictions of chemical shifts for 2-dimensional chemical structures have been used for decades, with the additivity rules exemplified by Pretsch^1^ and HOSE-code^2^ variants forming the basis of many analyses. However their applicability is limited by being based on 2-dimensional structures and cannot readily deal with 3-dimensional conformational or stereochemical analysis. Some modifications to treating 3-dimensional structures have been made by *e.g.* flat-but-stereochemically-aware HOSE codes^3^ or single conformer models of experimental systems^4^–^6^ but the improvements in 3-dimensional accuracy are limited as conformation and flexibility must necessarily be accounted for completely to achieve maximum accuracy. Multiple-bond ^1^H–^1^H coupling constants are more directly linked to 3-dimensional structure, however generically applicable Karplus-style empirical relationships, such as the widely used equation reported by Haasnoot *et al.*,^7^ suffer from lower accuracy when confronted with complex chemical functionality while equations designed for specific sub-structures, *e.g.* carbohydrates,^8^ are not applicable to the whole of chemical space. Finally, many NMR parameters, for example 1-bond ^1^H–^13^C scalar coupling constants, ^1^*J*_CH_, which are sensitive to both chemical connectivity and 3-dimensional structure are rarely used in isotropic studies precisely because there are no general fast predictive methods for ^1^*J*_CH_.

For all of these reasons, the accurate prediction of NMR parameters in modern 3-dimensional structure determinations relies increasingly on the use of quantum chemical calculations, typically based on Density Functional Theory (DFT).^9^–^12^ Optimal DFT methods can be accurate to within 1–2%, *e.g.*^1^*J*_CH_ predicted with <4 Hz accuracy to experiment^13^–^15^ (on values that range from roughly 100–250 Hz) and <0.2/<2 ppm^16^,^17^ (on ranges of ∼10/∼200 ppm) for ^1^H and ^13^C chemical shifts respectively. The substantial downside of DFT is the significant computation time required when using methods that can provide sufficient accuracy in NMR predictions. Accurate DFT-based predictions of chemical shift and scalar couplings typically take hours to days of CPU time for a single rigid molecule of even relatively low (∼500) molecular mass. The largest proportion of this CPU time is occupied by the NMR computations, especially when computing scalar coupling constants. Naturally, in cases where multiple conformers or isomers must be considered (and thus predictions for multiple structures are required) this becomes days to months of computation for a single study.

Machine learning methods offer a solution to the time-demands of DFT NMR predictions, achieving them in seconds rather than hours or days. Such machines, trained on experimental data, for ^1^H and ^13^C chemical shifts based on 2-dimensional structures are well-established.^18^–^21^ These systems are trained on hundreds of thousands of validated experimental chemical shifts arising from tens of thousands of chemical structures. Training such machines for prediction of scalar couplings is more challenging because accurate and validated experimental databases do not exist on this scale (*e.g.*^1^*J*_CH_ values) and they can be critically dependent on 3-dimensional structure (*e.g.*^3^*J*_HH/CH_ values). On the other hand, a machine could be trained using large datasets of DFT-computed NMR parameters, such as chemical shifts and scalar couplings, derived from 3-dimensional structures. Such large DFT-derived datasets can be generated systematically with minimal effort and are not limited to offering accuracy only for structures that are similar to previously experimentally determined molecules. With a large enough training database, such a machine would be expected to approach the accuracy of DFT calculation of NMR parameters for 3-dimensional structure analysis, but with several orders of magnitude reduction in time for the NMR predictions. This approach was recently reported for solid-state chemical shift predictions by Paruzzo *et al.* (SHIFTML,^22^) where the computational demand of DFT calculations on extended lattices are high and comparable to those needed for multi-conformer calculations on solution-state systems.

In this paper we describe the development of our first generation of solution-state NMR prediction machines – IMPRESSION (Intelligent Machine PREdiction of Shift and Scalar information Of Nuclei), trained on DFT-predicted values rather than relying on scarce or error-prone experimental data. We have chosen to demonstrate the versatility of machine learning of NMR parameters using both ^1^H and ^13^C chemical shifts and ^1^*J*_CH_ couplings. We include scalar couplings in addition to chemical shift, as the former are less amenable to machine learning based on experimental data, and ^1^*J*_CH_ precisely because it has been demonstrated to be valuable for elucidating both 2-dimensional connectivity and 3-dimensional structure^5^,^23^ but requires DFT to predict/interpret for most cases. Providing a fast and accurate predictive tool for ^1^*J*_CH_ will be especially valuable and could encourage wider acceptance of this and other accessible NMR parameters in structure determinations. We demonstrate that IMPRESSION can predict all these NMR parameters for organic molecules, including 3-dimensional discrimination, with up to DFT accuracy but several orders of magnitude faster and can be applied to experimental data with comparable outcomes to DFT.

## 2 Results and discussion

### 2.1 Dataset production and framework

In order to train and test IMPRESSION, we developed a dataset of NMR parameters (*δ*^1^H, *δ*^13^C, ^1^*J*_CH_), computed using DFT in the Gaussian09 software package.^24^ While more demanding computational methods could be considered,^25^ their computational cost would be extortionate with minimal improvement in outcomes for the training and testing datasets described. Instead we found that using mPW1PW91/6-311g(d,p) for optimisation and ωb97xd/6-311g(d,p)^26^–^30^ for computing the NMR parameters was computationally efficient and sufficiently accurate for comparison to experimental values across a range of NMR parameters. In the geometry optimisations a tight optimisation criteria and ultrafine integral grids were used to minimise molecular orientation affecting geometries and energies (see ref. 31 and references therein for a discussion of this). The NMR parameters were calculated using gauge independent atomic orbitals with uncontracted basis sets to improve descriptions of the core orbitals^30^ and calculation of all components of the scalar couplings (Fermi contact, spin dipole, diamagnetic spin orbit, paramagnetic spin orbit). The calculated magnetic shielding tensors were converted into chemical shifts using the linear scaling method and reference compounds reported by Tantillo *et al.*^10^,^32^ A training set of 882 structures (17 222 ^1^*J*_CH_; 18 383 *δ*^1^H; 17 081 *δ*^13^C values/environments) were selected by an adaptive sampling (active learning) procedure^33^–^35^ from a superset of 75 382 chemical structures comprising only C, H, N, O and F atoms in the Cambridge Structural Database^36^ (accessed 7/9/2018). The adaptive sampling procedure trains an initial IMPRESSION machine from 100 chemical structures and then uses this machine to predict the parameters for all remaining structures in the superset to measure their variance in a 5-fold cross validation (*i.e.* how much a given parameter changes when predicted from 5 separate machines each trained on a different 80% subset of the current training set). The 100 structures in the superset which show the highest variance are then added to the training dataset and the cycle is iterated (see ESI for further details†). Adaptive sampling therefore adds the 100 structures at each training iteration which IMPRESSION is the most uncertain about. In doing so, each added structure provides the maximum benefit to the machine and substantially reduces the overall computational cost required to reach a given accuracy. The test set, against which the quality of the IMPRESSION predictions is independently tested, was comprised of a further 410 chemical structures (7788 ^1^*J*_CH_; 7832 *δ*^1^H; 7522 *δ*^13^C environments) harvested from the CSD-500 dataset recently reported by Paruzzo *et al.*^22^

IMPRESSION uses a Kernel Ridge Regression^37^ (KRR) framework to learn the ^1^*J*_CH_ scalar couplings and ^13^C and ^1^H chemical shifts of molecular structures. KRR was successfully used by Paruzzo *et al.* to develop SHIFTML.^22^ Neural networks have also been used to predict chemical shifts in small molecules from experimental data,^6^,^38^,^39^ however we found no clear advantages in using feed forward neural networks in this work as the accuracy was comparable to KRR for the datasets used, with the kernel methods being much faster to train with the given training set size. In order to encode the similarity between chemical environments of each molecular structure we tested three approaches previously described – Coulomb matrices,^40^ aSLATM,^41^ and FCHL^42^ all available from the QML python package.^43^ We refer the reader to Section S1.1 in the ESI† and the respective papers describing each representation for more details. All of these kernel similarity measures compare *atomic* environments, so in the case of ^1^*J*_CH_, we used the product of the separately calculated kernel similarities for the ^1^H and ^13^C nuclei as this performed better than either atomic environment alone. The KRR procedure is further described in the ESI (Section S1.1†).

**Fig. 1:**
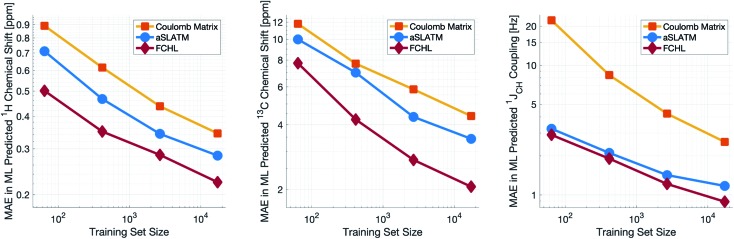
log–log plot of training set size *vs.* the mean absolute error between ML predictions and DFT of the test set for *δ*^1^H (left), *δ*^13^C (centre) and ^1^*J*_CH_ couplings (right). Results are shown for the Coulomb matrix, aSLATM and FCHL kernel similarity measures.

Both aSLATM and FCHL were found to outperform Coulomb matrices (Fig. 1), which is expected as Coulomb matrices only include 2-body interactions, while aSLATM and FCHL both include three-body interactions as well. As FCHL provided the best performance for all three parameters and was substantially more computationally efficient than aSLATM, it was used in the final development of the full IMPRESSION machine.

### 2.2 Performance relative to DFT

**Fig. 2:**
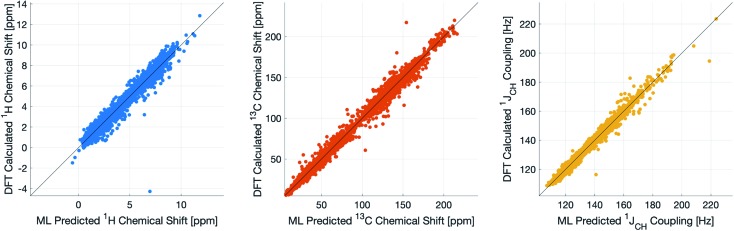
IMPRESSION machine learning predictions compared to DFT computed NMR parameters for *δ*^1^H (left), *δ*^13^C (centre) and ^1^*J*_CH_ couplings (right) without variance filtering.

During training, the machine performance for prediction of all NMR parameters (*δ*^1^H, *δ*^13^C, ^1^*J*_CH_) improved steadily with increasing training set size, as illustrated in the learning curves (Fig. 1). This indicates that the accuracy of the machine can be further improved by adding additional training data, however the absolute gains become marginal beyond the dataset size used here with a ten-fold increase in training set size approximately halving the average error between IMPRESSION and DFT. After training on the full set of 882 chemical structures, IMPRESSION predictions achieved mean absolute errors (MAE) of 0.23 ppm/2.45 ppm/0.87 Hz for *δ*^1^H/*δ*^13^C/^1^*J*_CH_/predictions and root mean squared error (RMSE) of 0.35 ppm/3.88 ppm/1.39 Hz against the independent test set (Fig. 2).

Notably however, a very small number of predictions for the test set were much less reliable. For example, 186 (∼2.3%) of the *δ*^1^H values had errors >1 ppm between IMPRESSION and DFT, with a maximum error (MaxE) of 11.22 ppm. Similar outcomes were observed for the other parameters with 187 *δ*^13^C values (∼2.5%) with errors >10 ppm (MaxE = 63.33 ppm) and 14 (∼0.2%) of the 7788 predicted ^1^*J*_CH_ values having errors of >10 Hz (MaxE = 24.63 Hz). Diagrams of the structures containing the five most significant outliers for each NMR parameter are shown in Fig. S19–S21 in the ESI.† Examination of the chemical environments of the most significant outliers show that they arise from unusual functional groups such as those containing sp-hybridised atoms, or unusual 3-dimensional environments such as atoms near pi-systems of aromatic rings. These outliers suggest that, as desired, the machine learning system is indeed very sensitive to the 3-dimensional relationships of the atoms in the structure. However this same sensitivity also makes IMPRESSION less accurate for chemical environments which are not very similar to environments across the 882 molecular structures used to train IMPRESSION.

**Fig. 3:**
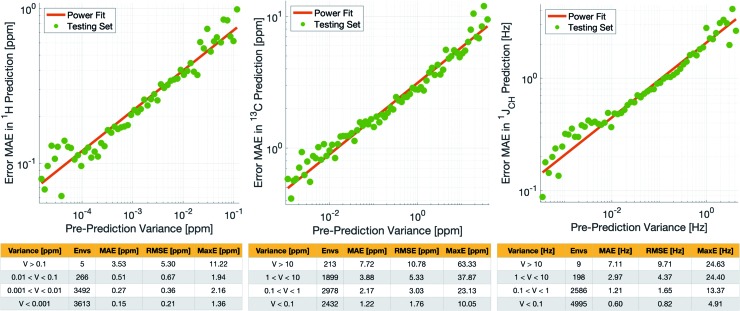
(Top) Correlation between pre-prediction variance and prediction error between DFT and IMPRESSION for *δ*^1^H (left), *δ*^13^C (centre) and ^1^*J*_CH_ couplings (right) on the test set. The prediction errors were binned by variance and an average error (MAE) was produced for each bin. (Bottom) Error metrics for different variance ranges.

Crucially, we are able to *a priori* identify poorly described environments using the same variance-based approach used to generate the training set. By assessing the variance in the prediction of a given NMR parameter across a 5-fold cross-validation, we can quantify our confidence in each individual prediction since environments which are poorly described by the chemical structures in the training set will have high variance in this cross-validation. There is indeed a clear correlation of variance against prediction error for the independent test set (Fig. 3). The tables in Fig. 3 suggest that the bulk of the environments are predicted very accurately, and that the high variance environments are the dominant source of the large outliers.

In principle, removing IMPRESSION-predicted values which show high variances in cross-validation should provide a “pre-prediction variance filter” that will substantially improve the quality of, and thus the confidence in, IMPRESSION predictions. Selecting an appropriate variance cut-off for each NMR parameter is then simply a balance between desired prediction quality and the number of predictions which will be excluded by that cut-off. Reports of DFT accuracy with respect to experiment for ^1^H and ^13^C chemical shift predictions vary significantly, but typically in the range of 0.2–0.4 ppm/2–4 ppm, with the best reported accuracies down to <0.2/<2 ppm (ref. 16 and 17) in optimal cases. Similarly, Buevich *et al.* recently highlighted that current best-in-class DFT methods predict ^1^*J*_CH_ experimental values with accuracies of 2–4 Hz, when presenting an optimised workflow for calculating ^1^*J*_CH_ values which achieved an RMSE of 1.61 Hz.

We therefore identified variance cut-offs for IMPRESSION predictions that provide a good compromise between accuracy and excluded values for the test set, which were found to be 1 Hz for ^1^*J*_CH_, 0.1 ppm for *δ*^1^H and 5 ppm for *δ*^13^C. Applying these pre-variance filter values improves the fits between IMPRESSION and DFT to levels that are comparable with literature reports for MAE/RMSE of DFT *vs.* experiment (MaxE is rarely reported for large experimental validations, but the reader can find comparators from our experimental validations described below in Section 2.3). For *δ*^1^H the 0.1 ppm filter excludes 5 environments (<0.1%) and improves the fit to MAE = 0.23 ppm, RMSE = 0.32 ppm; MaxE = 2.16 ppm. For *δ*^13^C a 5 ppm filter provided a good fit (MAE = 2.17 ppm; RMSE = 3.25 ppm; MaxE = 37.87 ppm) while excluding 538 (∼7.2%) of the environments. For ^1^*J*_CH_ a 1 Hz filter improved the fit to MAE = 0.81 Hz, RMSE = 1.17 Hz; MaxE = 13.37 Hz while discarding only 207 (<3%) of the environments.

As highlighted by the learning curves, further improvement to the machine predictions of DFT NMR results can be made by increasing the size of the DFT-derived training dataset by around an order of magnitude. However at this stage variance-filtered IMPRESSION compares well enough with respect to DFT that it was taken forward. It should also be noted at this point that IMPRESSION only accelerates NMR prediction, it does not accelerate the 3D structure generation by DFT (which can still take hours/days). This overall time, *i.e.* 3D structure generation + NMR prediction, could be reduced further by using 3D structures derived from molecular mechanics rather than DFT. While not the key focus here, the use of molecular mechanics structures as inputs to a re-trained IMPRESSION machine was explored. While practical, this resulted in a ∼30–50% increase in the average prediction errors for *δ*^1^H and ^1^*J*_CH_ presumably arising from a mismatch between the detail of molecular mechanics geometries and those used to calculate the DFT NMR parameters (see Section S2 in the ESI for details†). Interestingly, *δ*^13^C predictions were relatively insensitive to this change, perhaps reflecting better description of carbon environments by molecular mechanics forcefields. This is an exciting avenue to explore further, but to focus the discussion here on the ability of IMPRESSION to reproduce DFT NMR predictions, the subsequent experimental comparisons are based on the IMPRESSION machine trained on the same DFT-geometries used for the DFT NMR predictions.

### 2.3 Performance relative to experiment

Naturally, a key test of IMPRESSION is its ability to reproduce DFT predictions of experimental values of relevant compounds. To test this for ^1^*J*_CH_, a validation set of 608 experimental ^1^*J*_CH_ values were taken from structures collated by Venkata *et al.*^23^ which contain C, H, N, O and F elements only. Firstly, we checked the ability of our ωb97xd/6-311g(d,p) DFT method itself to reproduce these experimental results. It should be noted in the subsequent analysis that all DFT and IMPRESSION predictions were based on the single conformers that Venkata *et al.* reported for each compound. While not making the predictions entirely experimentally relevant, it allows direct comparison between DFT and IMPRESSION NMR predictions for this data. Calculating the 608 couplings with ωb97xd/6-311g(d,p) took 156 CPU hours and initially gave a relatively poor fit to experiment (MAE = 10.92 Hz) but with a systematic offset from the experimental data by an average of –10.91 Hz. Adding this systematic offset to the DFT-predicted values provided a good fit between DFT and experiment (MAE = 2.16 Hz; RMSE = 3.33 Hz; MaxE = 20.05 Hz) and this was used for all subsequent comparisons to experiment based on this DFT method. As IMPRESSION is trained on DFT data computed with this same ωb97xd/6-311g(d,p) method and both methods use only single conformer predictions for each molecule, then these statistics represent a practical limit for the accuracy that we might expect from IMPRESSION on this experimental data.

**Fig. 4:**
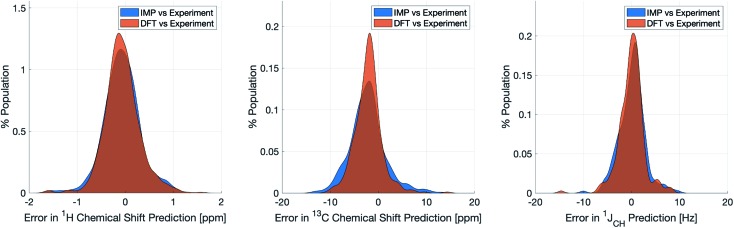
Distribution of errors for machine learning NMR predictions and DFT calculations when compared to the relevant experimental validation dataset for *δ*^1^H (left), *δ*^13^C (centre) and ^1^*J*_CH_ couplings (right). Variance filters applied to IMPRESSION predictions: *δ*^1^H = 0.1 ppm (0 of 734 environments removed), *δ*^13^C = 5 ppm (24 of 457 environments removed), ^1^*J*_CH_ = 1 Hz (143 of 608 environments removed).

IMPRESSION took only 60 CPU seconds to predict the full set of 612 ^1^*J*_CH_ values but with some substantial outliers (MAE = 4.52 Hz; RMSE = 10.49 Hz; MaxE = 120.3 Hz). Applying the 1 Hz variance filter gave: MAE = 2.01 Hz, RMSE = 2.69 Hz, MaxE = 10.01 Hz (removing 143 values) which was essentially identical accuracy to that obtained from the DFT method for these same filtered environments: MAE = 1.83 Hz, RMSD = 2.60 Hz, MaxE = 14.63 Hz. An overlay of the error distributions for DFT and the 1 Hz variance-filtered IMPRESSION *vs.* the experimental values (Fig. 4) demonstrates the comparability between machine learning and DFT for ^1^*J*_CH_ predictions. This represents quite excellent performance of the machine for reproducing experimental data in just a few seconds, with quality for the majority of environments as good as the best MAEs (1.5–4 Hz) described by Buevich *et al.* as typical for DFT methods, with <25% of the values being tagged as unreliable by the variance filter. Of course, if a slight loss in prediction quality is acceptable for a given study, then more predicted values could be retained by using a slightly looser variance-filter.

Similar accuracy could be obtained for IMPRESSION predictions of 734 ^1^H chemical shifts for 36 structures reported by Smith and Goodman^44^ in their DP4 dataset (again, single conformers were used for both DFT and IMPRESSION predictions). IMPRESSION predictions gave MAE = 0.29 ppm, RMSD = 0.38 ppm, MaxE = 1.59 ppm with a variance filter of 0.1 ppm but in this case no environments were removed with the variance filter and provided essentially the same outcomes as the ωb97xd/6-311g(d,p) DFT method on the same single conformer structures (MAE = 0.28 ppm, RMSE 0.37 ppm, MaxE 1.62 ppm, see Fig. 4 for an overlay of errors). The IMPRESSION predictions for *δ*^13^C using the 5 ppm variance filter identified during training and testing of the machine compared slightly less well to the DP4 experimental dataset (MAE = 3.44 ppm, RMSE = 4.30 ppm, MaxE = 13.06 ppm, removing 24 environments) than DFT (MAE = 2.78 ppm, RMSE = 3.48 ppm, MaxE = 14.33 ppm). A tighter 1 ppm variance filter for the *δ*^13^C predictions was examined, but gave only a slight improvement in prediction quality MAE = 3.20 ppm, RMSE = 4.00 ppm, MaxE = 13.03 ppm while removing 120 out of the 458 carbon environments.

At every stage in this study we found that the IMPRESSION *δ*^13^C predictions have a wider distribution of errors than the other NMR parameters when compared to the quality of the DFT from which they are trained. This is unsurprising given that the structural environments of ^13^C nuclei in molecules are inherently more complex than ^1^H given the higher valency and thus more complex bonding environments and geometries, so in future development, larger training datasets focussed on optimising *δ*^13^C predictions will be beneficial.

### 2.4 3-Dimensional structure discrimination

**Fig. 5:**
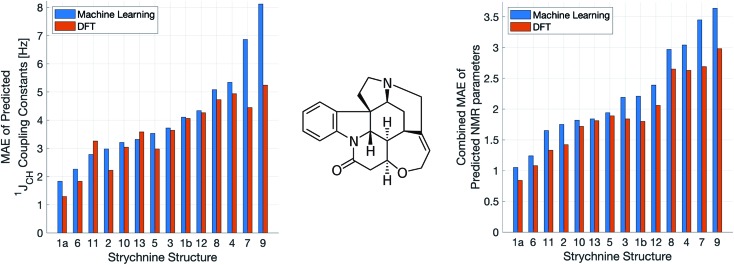
Errors from comparison of NMR experimental data of the natural product strychnine (centre) to IMPRESSION (blue) and DFT (red) predictions for 13 diastereomers of strychnine, including two conformers for the natural product **1**: the lowest energy **1a** (>97% populated) and the next lowest energy **1b** (<3% populated). The left hand plot shows MAE for ^1^*J*_CH_ while the right hand plot shows the geometric mean absolute error for all NMR parameters (*δ*^1^H, *δ*^13^C and ^1^*J*_CH_) combined. Variance filters applied to predictions: *δ*^1^H = 0.1 ppm, *δ*^13^C = 5 ppm, ^1^*J*_CH_ = 1 Hz.

A demanding test of IMPRESSION is in its ability to predict and discriminate experimental NMR data for stereoisomeric compounds *i.e.* those that differ only in their 3-dimensional structure, but not connectivity. Even though IMPRESSION has not been explicitly trained to deal with multiple conformers/isomers of any one compound, 3-dimensional variation is implicit within the varied chemical structural space of the adaptively sampled training set. Buevich *et al.* recently demonstrated^5^ that DFT prediction of ^1^*J*_CH_ values can successfully discriminate the naturally occurring structure **1** of the polycyclic alkaloid strychnine (Fig. 5, centre) from 12 other diastereomers (see ESI Section S5† for the structures) based on comparison with the experimental ^1^*J*_CH_ values of the natural product. Pleasingly, the same test conducted with IMPRESSION-predicted ^1^*J*_CH_ values (blue bars in Fig. 5, left) also correctly identifies the natural product diastereomer **1a** as having the smallest error (MAE = 1.87 Hz; RMSE = 2.50 Hz; MaxE = 6.19 Hz). The error for the correct structure is ∼30% lower than the diastereomer with the second lowest error **6** (MAE = 2.48 Hz; RMSE = 3.38 Hz; MaxE = 8.42 Hz) and this is very similar to the discrimination offered by ωb97xd/6-311g(d,p) (red bars in Fig. 5). Indeed IMPRESSION could also distinguish between the 3-dimensional structures of **1a**, the lowest energy conformer of the natural product (97% population in solution), and **1b** which is the second lowest energy conformer (3% population in solution).^45^ So while the absolute accuracy of IMPRESSION for predicting ^1^*J*_CH_ values for strychnine (MAE = 1.87 Hz) is slightly lower than that obtained from the DFT method (MAE = 1.31 Hz), its discriminating power between structural isomers is nearly the same.

Combining IMPRESSION predictions for ^1^*J*_CH_ with ^1^H and ^13^C chemical shifts also provides correct identification of the naturally occurring structure, but IMPRESSION and DFT now both see structure **2** as the next best candidate (Fig. 5, right). This is due to the experimental *δ*^1^H values having better agreement with the predictions for diastereomer **2** than **1a** for DFT and also IMPRESSION. While this is obviously problematic for structure elucidation purposes, it clearly arises because of a deficiency in the DFT prediction of ^1^H chemical shifts, which is then faithfully reproduced by IMPRESSION. For the individual MAE values across all three parameters see ESI Section S5.†

Similarly, we found that IMPRESSION predictions can be used to correctly assign the diastereotopic protons in strychnine. IMPRESSION and DFT predictions of ^1^*J*_CH_ for the diastereotopic protons in strychnine were consistently in line with each other (details can be found in Section S4 of the ESI†) and for the three methylene groups where there is a significant difference (≫2 Hz) in experimental ^1^*J*_CH_ values both methods correctly assign these protons (Fig. S16†).

Finally, we validated IMPRESSION chemical shift predictions for natural product structures. We conducted DFT and IMPRESSION predictions on structures from a recent report which suggested structural reassignments for oxirane-containing natural products on the basis of DU8+ DFT calculations.^46^ To avoid complications with incorrect DFT prediction of conformer energies leading to poor population averaging of NMR parameters from the constituent conformers, we limited the validation to ‘rigid’ structures in the report that contained only one dominant conformer after conformational searching. Pleasingly, while our results did not always agree with the DU8+ analysis, IMPRESSION was just as effective as our underlying ωb97xd/6-311g(d,p) DFT method in discriminating each original and revised chemical structure (see Section S3 in the ESI for more details†). Once again this confirms that IMPRESSION is capable of making predictions that are of comparable quality to it's underlying DFT method ωb97xd/6-311g(d,p), and thus any improvements in the DFT method used to train IMPRESSION will be subsequently expressed in the quality of IMPRESSION predictions.

## 3 Conclusions

In summary, this first generation IMPRESSION machine, trained on DFT-computed NMR parameters derived from a set of 3-dimensional structures is capable of reproducing DFT-predicted NMR parameters for a range of experimentally relevant systems with high accuracy but in a fraction of the time. Accurate and generalised prediction of NMR parameters for 3-dimensional applications has not been addressed by previous machine learning systems but the confidence provided by the variance-filtered IMPRESSION results makes this tool essentially as robust for 3-dimensional applications to experimental systems as DFT. At this stage, the two primary sources of error in IMPRESSION predictions of experimental data are errors in the underlying DFT method on which it is trained (of which there can be several^47^–^49^) and the range of chemical space covered by the current IMPRESSION training set. We are working to improve both of these factors, as well as extending the predictions to multiple-bond scalar couplings for future generations of IMPRESSION, along with developing a more rigorous statistical treatment of the predicted values taking into account the pre-prediction variance.

## Conflicts of interest

There are no conflicts to declare.

## Supplementary Material

Supplementary information
