# Supplementary material for: IMPRESSION – prediction of NMR parameters for 3-dimensional chemical structures using machine learning with near quantum chemical accuracy
Source: Chem Sci. 2019 Nov 20;11(2):508–15. doi: 10.1039/c9sc03854j (PMC7067266; doi:10.1039/c9sc03854j)
Supplement: Supplementary file 1 [file SC-011-C9SC03854J-s001.pdf]

Electronic Supplementary Material (ESI) for Chemical Science.
This journal is © The Royal Society of Chemistry 2019

Supplementary Information for IMPRESSION - Prediction of NMR Parameters for 3-dimensional chemical structures using Machine Learning with near quantum chemical accuracy

Will Gerrard, Lars Andersen Bratholm, Martin Packer, Adrian Mulholland, David Glowacki, Craig Butts

Contents

S1 Methods **2**
S1.1 Kernel ridge regression . . . 2
S1.2 Training and test data . . . 2
S1.3 Correction of DFT NMR parameter predictions for comparison to experiment . . . 3
S1.4 Adaptive sampling . . . 3
S1.5 Hyper-parameter optimisation . . . 3

S2 IMPRESSION performance using Molecular Mechanics geometries **4**
S2.1 DFT trained model . . . 4
S2.2 MMFF94 trained model . . . 5
S2.3 Computational timings . . . 6

S3 Structure revision examples **7**
S3.1 Geometric mean for diastereomer discrimination . . . 7
S3.2 Crithmifolide . . . 7
S3.3 Caespitenone . . . 8
S3.4 Secoafricane . . . 9
S3.5 Grandilobalide B . . . 10
S3.6 Toluene dioxide . . . 11

S4 Diastereotopic proton assignment in Strychnine **12**

S5 Strychnine diastereomers **14**

S6 Large errors **18**
S6.1 ^{1}H chemical shifts . . . 18
S6.2 ^{13}C chemical shifts . . . 19
S6.3 $^{1}J_{CH}$ coupling constant . . . 20

S7 Gaussian input files **21**

S8 CSD structures **22**

S9 Full Gaussian reference **27**

S1 Methods

S1.1 Kernel ridge regression

Kernel Ridge Regression[1] (KRR) provides a systematic way to map geometric features of a chemical environment (i.e. the chemical identity and geometry of atoms in the environment surrounding atoms of interest) to a target observable (in this case scalar coupling constants or chemical shifts), effectively interpolating between known data points. The observable of interest (y_i) for a given environment ($\mathbf{E}_i$) is estimated as a linear combination of it's similarity to the environments ($\mathbf{E}_j$), for which the corresponding observable is known:

$$y_i^{\text{pred}} = \sum_j^N \alpha_j k\left(\mathbf{E}_i, \mathbf{E}_j\right), \tag{1}$$

Here N is the number of chemical environments in the training data set and k is a kernel function that computes the similarity between two environments. The kernel function typically takes a value of 1 for identical environments and approaches asymptotically 0 when environments become increasingly different. The regression parameters $\boldsymbol{\alpha}$ are regression coefficients that can be fitted to the training data by regularized least-squares optimization:

$$\underset{\boldsymbol{\alpha}}{\text{minimise}} \sum_i^N \left(y_i^{\text{exp}} - y_i^{\text{pred}}\right)^2 + \lambda \sum_i^N \alpha_i^2, \tag{2}$$

where y_i^{pred} is given by equation (1). λ controls the strength of the l_2-regularization, which is a penalty term to the loss function that favours the regression coefficients to be more uniform and to take smaller values. This effectively reduces overfitting and if properly tuned can improve transferability to new chemical environments.

Several functional forms of the kernel similarity measure has been proposed in recent years. In this work we compare three different kernel functions. The atomic Coulomb Matrix[2] was one of the early successful vector representations of the chemical environment around an atom and includes two-body interactions (distances) between a given atom and all atoms within a specified cutoff radius. The Atomic Spectral London Axilrod-Teller-Muto[3] (aSLATM) representation is a separate approach that also includes three-body interactions (angles). Both representations generate a vector ($\boldsymbol{x}$) per environment, where the kernel similarity can computed with a laplacian kernel:

$$k\left(\mathbf{E}_i, \mathbf{E}_j\right) = \exp\left(-\frac{\|\boldsymbol{x}_i - \boldsymbol{x}_j\|_1}{\sigma}\right), \tag{3}$$

where the kernel width σ determines how quickly the similarity measure converges towards 0.
FCHL[4] (acronym derived from the authors surnames) also includes three-body terms, but generates the kernel similarity directly, rather than through an intermediate vector representation step.

Since the above kernel similarity measure indicates how similar the chemical environment around two *atoms* are, we chose to use the product of the kernel similarity between the two hydrogens and the two carbons to represent $^1J_{CH}$ environments:

$$k\left(\mathbf{E}_i^{\text{CH}}, \mathbf{E}_j^{\text{CH}}\right) = k\left(\mathbf{E}_i^{\text{H}}, \mathbf{E}_j^{\text{H}}\right) k\left(\mathbf{E}_i^{\text{C}}, \mathbf{E}_j^{\text{C}}\right), \tag{4}$$

where $\mathbf{E}_i^{\text{CH}}$ is the joint set of chemical environments around the hydrogen and carbon atom involved in the scalar coupling. Alternatively just the kernel similarity between hydrogen atoms could be used, but we found an improvement in performance by also including the carbon similarity.
All representations and kernels as well as optimisation of the regression parameters were performed with the QML python library[5].

S1.2 Training and test data

The KRR machine was trained using 17,222 coupling environments from 882 chemical structures selected by adaptive sampling (active learning)[6–8] from the Cambridge structural database (filtering first for structures that contain only C, H, N, O and F elements, see section S1.4 for details) then optimising the structures and calculating the DFT NMR parameters (see next paragraph for details). The test set contained an independent set of 7832 environments from 410 chemical structures from the randomly selected CSD-500 test set reported by Emsley et al[9]. All DFT calculations were carried out using the Gaussian09 Rev. D software package[10] (See section S7 for example input files). The 3-dimensional chemical structures were each optimised with *mPW1PW91*[11]/6-311g(d,p)[12,13] using tight optimisation criteria and ultrafine integral grids were used to minimise molecular orientation affecting geometries and energies (see reference[14] and references therein for a discussion of this) and the resulting optimised structures were used to compute NMR parameters with *ωb97xd*[15]/6-311g(d,p). The NMR computations used gauge independent atomic orbitals and were conducted with an uncontracted basis set for coupling calculations[16], called with the 'mixed' option within the Gaussian09 software. The scalar coupling values obtained from the calculations included all terms calculated: Fermi contact, spin-dipolar, paramagnetic spin-orbit and diamagnetic spin orbit terms are all included in the total nuclear spin-spin coupling produced in the output files. Some DFT structure optimisations failed to converge to an energy minima and these were excluded from the final datasets.

S1.3 Correction of DFT NMR parameter predictions for comparison to experiment

The DFT calculated magnetic shielding tensors were converted to chemical shifts using a linear scaling method and reference compounds reported by Tantillo *et al*[17,18]. The results of this linear scaling are shown in figure S1.

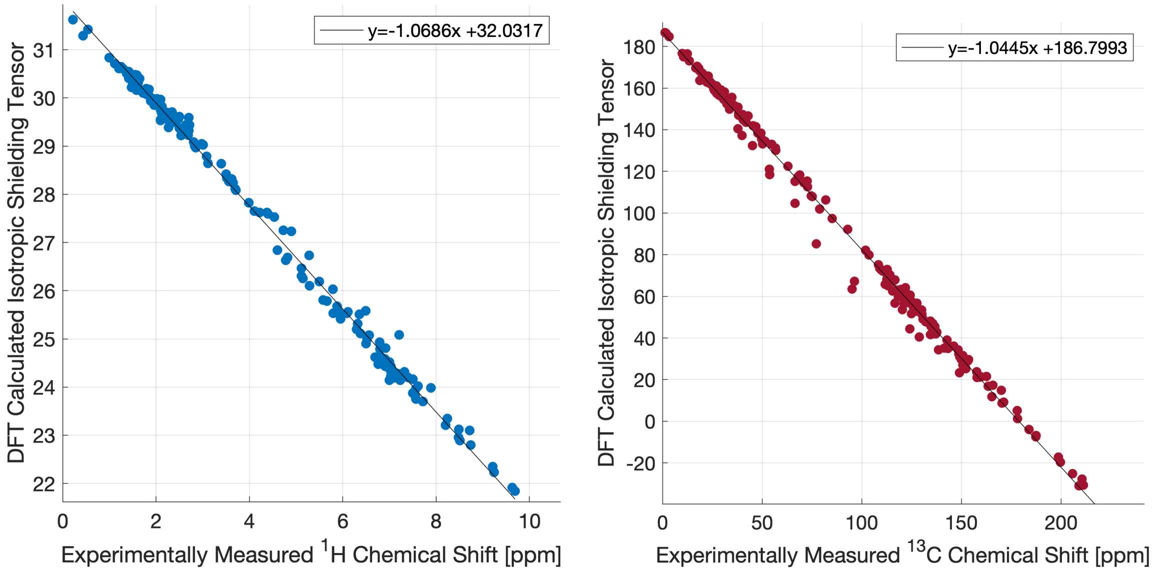

Figure S1 Calculation of tantillo regression scaling factors for a) δ^{1}H and b) δ^{13}C

For the $^{1}J_{CH}$ data used in this work, a clear linear offset was found upon comparison of the DFT values to experimentally measured data. As a result, the offset (10.91Hz) was applied to the DFT values in both the training and test datasets.

S1.4 Adaptive sampling

The training set was obtained via an adaptive sampling approach. An initial set of 100 structures were chosen at random from the CSD-500 test set already obtained from the work by Emsley et al[9]. 5 subsets of 80 structures each were then used to train separate models to predict $^{1}J_{CH}$ coupling constants, ^{1}H and ^{13}C chemical shifts for all organic structures in the Cambridge Structural Database containing only H/C/N/O/F atoms. The variance in the predictions of the five models (pre-prediction variance) is a measure of how confident one can be in a given prediction. 300 structures containing the environments with the highest variance were selected to be added to the training set (100 each based on the $^{1}J_{CH}$, ^{1}H and ^{13}C variance). Structure optimizations and NMR computations were performed for these to build the training set. The initial random set of 100 structures was discarded after the first round, and the process was repeated four times. Some structures failed to optimise in each round and were discarded leading to a training set consisting of 882 structures.

S1.5 Hyper-parameter optimisation

The following hyper-parameters were optimized for the machine learning procedure: the cutoff radius, the kernel width and the l2-regularisation factor. The optimal combination of these three variables was found through a cross-validated gaussian-process led search using the python module BayesianOptimization[19]. The optimal parameters were determined as those with the lowest average mean absolute deviation across a five-fold cross-validation using the training set environments.

S2 IMPRESSION performance using Molecular Mechanics geometries

Whilst the focus of this work is to develop a machine learning method to replace the DFT calculation of NMR parameters, the geometry optimisation used in preparing the structures in all datasets still accounts for 26% of the total CPU time. The effect of replacing the DFT geometry optimisation step with a molecular mechanics based optimisation was investigated through two methods.

S2.1 DFT trained model

Firstly, the existing models (trained using DFT optimised geomtries) were used to make predictions on structures optimised through the MMFF94 forcefield[20]. The result was a decrease in accuracy of all three models but especially so for $1J_{CH}$ and δ^{13}C. The error distributions in figure S2 show a reduction in the quality of the predictions on all three parameters.

	MAE	RMSE	MaxE	Variance Cutoff	Envs removed
δ^{1}H	0.26ppm	0.38ppm	5.55ppm	0.1ppm	1
δ^{13}C	3.30ppm	4.63ppm	37.42ppm	5ppm	949
$^{1}J_{\mathrm{CH}}$	2.30Hz	3.00Hz	20.44Hz	1Hz	5009

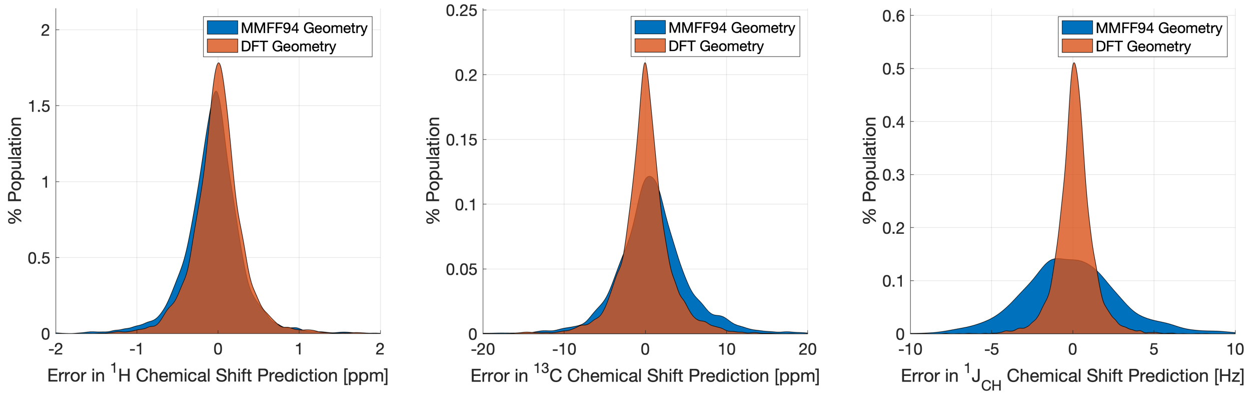

Figure S2 Error distributions for IMPRESSION predictions of molecular mechanics structures, using IMPRESSION models trained using DFT geometries. Variance filters applied: δ^{1}H = 0.1ppm, δ^{13}C = 5ppm, $^{1}J_{\mathrm{CH}}$ = 1Hz.

S2.2 MMFF94 trained model

Additionally, new models were trained based on molecular mechanics optimised training structures. The entire set of training and testing structures were reoptimised using the MMFF94[20] forcefield. These structures were associated with the previously calculated DFT NMR parameters and used to train and test new models. The model hyper-parameters were optimised using the same method as the DFT trained models and achieved an accuracy which was up to 50% worse than the models trained using DFT optimised structures.

	MAE	RMSE	MaxE	Variance Cutoff	Envs removed
δ^1H	0.28ppm	0.40ppm	5.20ppm	0.1ppm	3
δ^{13}C	2.31ppm	3.48ppm	39.92ppm	5ppm	952
$^1J_{CH}$	1.19Hz	1.75Hz	20.40Hz	5Hz	143

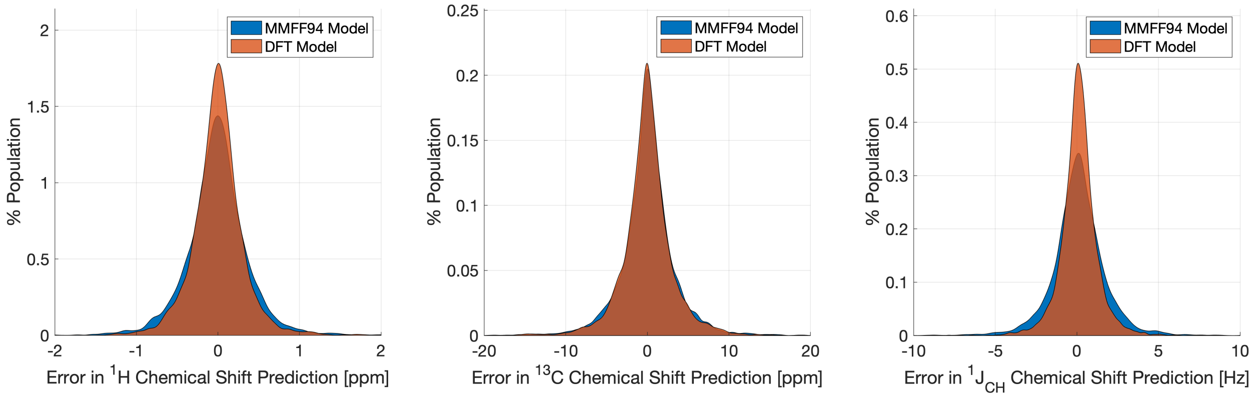

Figure S3 Error distributions for models trained using MMFF94 geometries predicting on structures with MMFF94 geometries, compared to the original DFT models from the main text. Variance filters applied: δ^1H = 0.1ppm, δ^{13}C = 5ppm, $^1J_{CH}$ = 1Hz.

S2.3 Computational timings

To highlight the value of replacing the NMR calculation with a machine learning solution, the distributions of CPU cost for all calculations in producing the training set are included here in figure S4. The 'mixed' option which uses an uncontracted basis set for calculating the fermi contact term is only relevant for coupling calculations so this has been removed from figure S4b. The mean CPU time for an optimisation was 15 hours across all 882 structures, whilst the mean CPU time for a DFT NMR calculation was 42 hours (or 22 hours without mixed). The use of a machine learning model to replace the NMR calculation therefore represents a significant time saving.

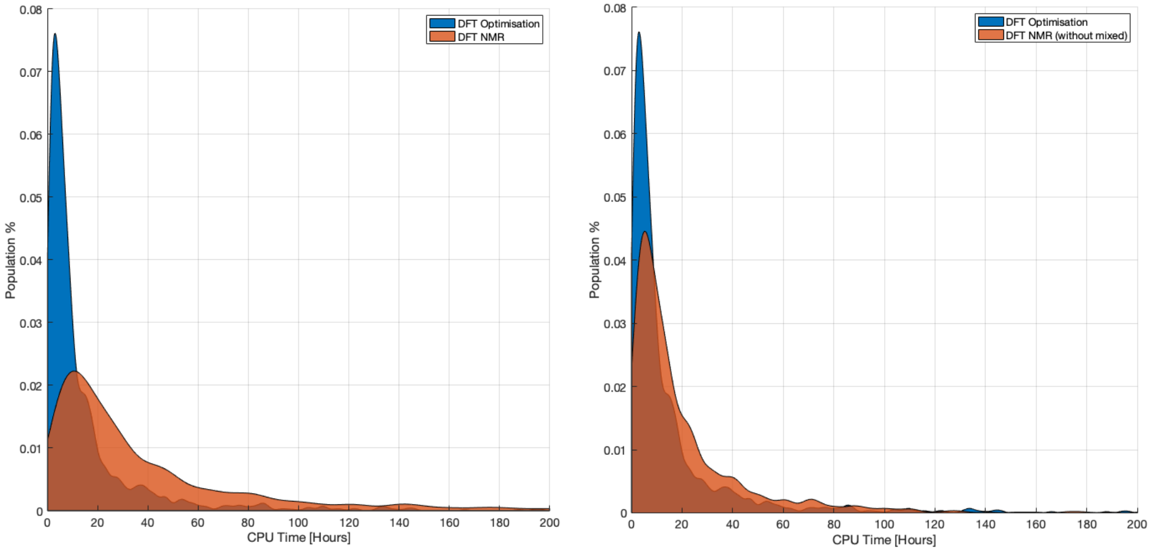

(a) (b)

Figure S4 Distribution of CPU time for DFT calculations on the training dataset. Mean time for optimisation = 15 Hours. a) with mixed option: Mean time for NMR calculation = 42 Hours. b) without mixed option: Mean time for NMR calculation = 22 Hours.

S3 Structure revision examples

To further demonstrate the applicability of the IMPRESSION predictions to structural elucidation problems, 5 examples of proposed natural product structure revisions from the literature were investigated[21]. No $^{1}J_{CH}$ values were reported for these compounds, so we can only make comparisons using the chemical shift models.

For the 5 compounds, Cartesian coordinates for the original and revised structures were obtained from the literature along with the experimental δ^{1}H and δ^{13}C assignments. The Cartesian coordinates were optimised, NMR parameters were computed and IMPRESSION predictions were made for each structure. The mean absolute error between the IMPRESSION predictions and experiment were compared to the corresponding MAE between the DFT calculations and the experimental values. Variance cutoffs of 1Hz, 0.1ppm, and 5ppm were used for $^{1}J_{CH}$, δ^{1}H, and δ^{13}C respectively.

S3.1 Geometric mean for diastereomer discrimination

As we combine different types of data to gather evidence for a given diastereomer, we take the geometric mean of mean absolute errors for each of the parameters:

$$\mathrm{MAE_{combined}} = \sqrt[3]{\mathrm{MAE}_{^{1}J_{\mathrm{CH}}}\mathrm{MAE}_{\delta^{1}\mathrm{H}}\mathrm{MAE}_{\delta^{13}\mathrm{C}}} \tag{5}$$

or in the case where $^{1}J_{CH}$ values are not avalable:

$$\mathrm{MAE_{combined}} = \sqrt[2]{\mathrm{MAE}_{\delta^{1}\mathrm{H}}\mathrm{MAE}_{\delta^{13}\mathrm{C}}} \tag{6}$$

S3.2 Crithmifolide

Comparing the results from our DFT method to that used in the original work, the predictions for the ^{1}H chemical shifts do not show the same improvement in accuracy between the original and revised structures. In the original work an improvement of 0.08ppm RMSE was reported, whereas comparisons using our DFT method found an increase in MAE of 0.03ppm (and RMSE of 0.01ppm). Pleasingly the IMPRESSION results mirror this discrepancy and match the DFT method on which the model was trained.

The ^{13}C chemical shift results from our DFT method agree with the literature, showing an improvement in fit from the original to the revised structure. The indecisive results from the geometric mean comparison reflect this discrepancy between the two chemical shift comparisons.

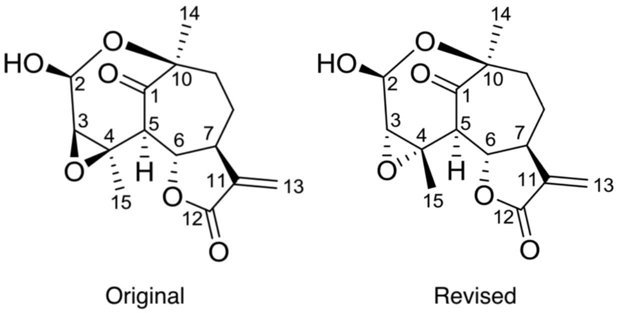

Figure S5 Original and revised structures for Crithmifolide.

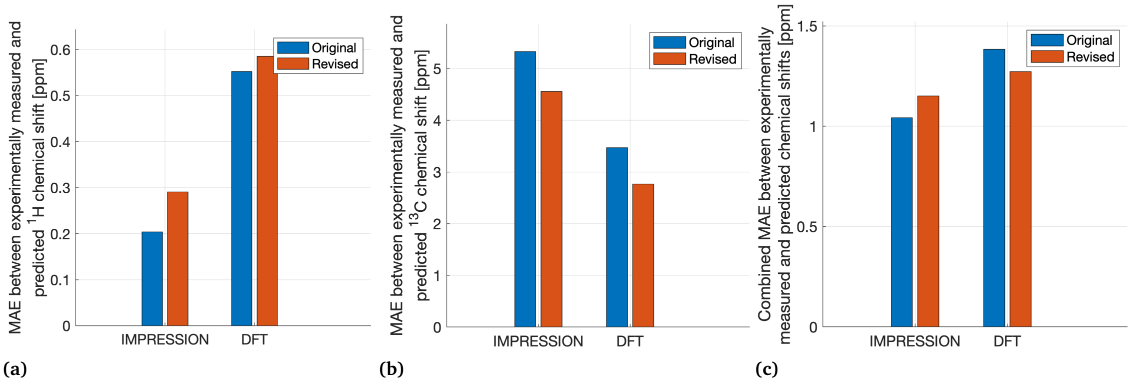

Figure S6 Change in the fit between prediction and experiment for both DFT and IMPRESSION for Crithmifolide. a) δ^{1}H. b) δ^{13}C. c) Geometric mean across both parameters.

S3.3 Caespitenone

The results for Caespitenone show good agreement between IMPRESSION, our DFT method, and the previously published results. Large deviations (>5ppm) were reported in the ^{13}C chemical shift results, and the DFT method used in this work reproduces this. The IMPRESSION predictions show the same change in the fit to experiment.

The methods used in this work also showed a significant improvement in fit for the ^{1}H chemical shifts, resulting in a reduction in error of over 50% for both our DFT method and IMPRESSION.

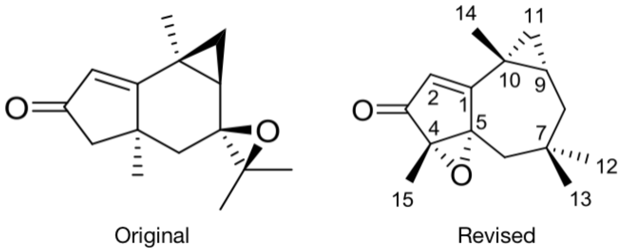

Figure S7 Original and revised structures for Caespitenone

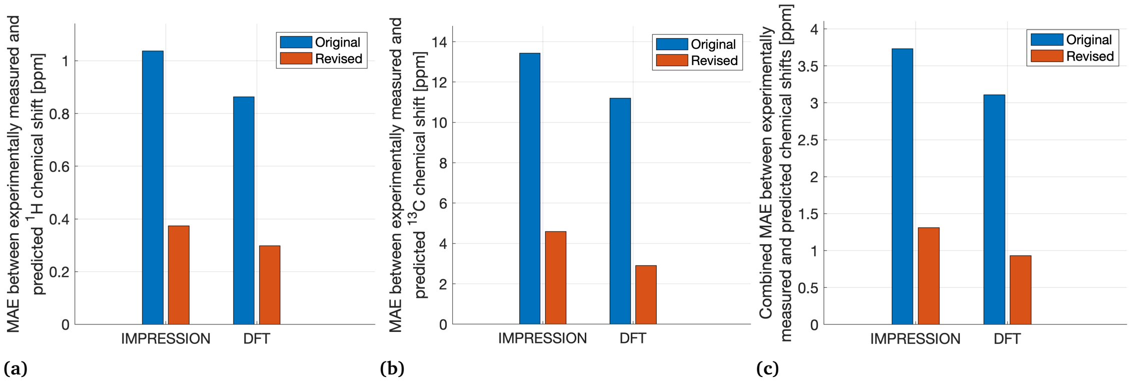

Figure S8 Change in the fit between prediction and experiment for both DFT and IMPRESSION for Caespitenone. a) δ^{1}H. b) δ^{13}C. c) Geometric mean across both parameters.

S3.4 Secoafricane

The reported values in the original work show a significant improvement in fit between experiment and calculation for both ^1H and ^{13}C chemical shifts. The results from our DFT method show a smaller but still significant improvement in fit for both parameters, and IMPRESSION mimics these results, but with a smaller change in MAE for the ^{13}C comparison.

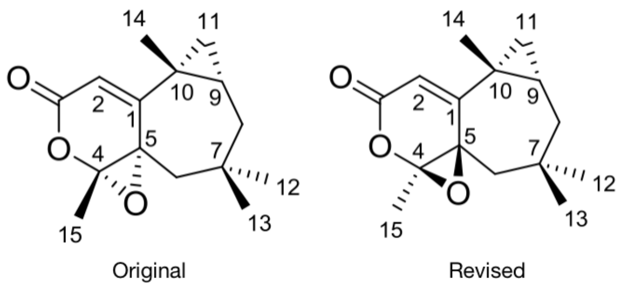

Figure S9 Original and revised structures for Secoafricane.

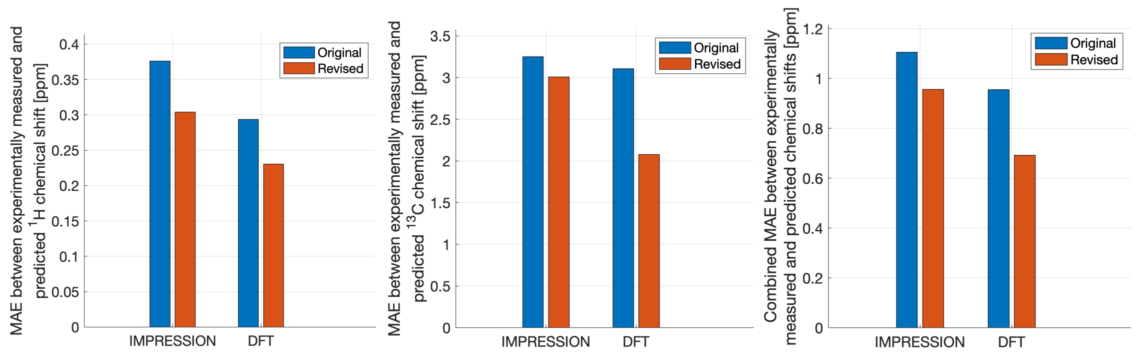

(a) (b) (c)

Figure S10 Change in the fit between prediction and experiment for both DFT and IMPRESSION for Secoafricane. a) δ^1H. b) δ^{13}C. c) Geometric mean across both parameters.

S3.5 Grandilobalide B

The literature results for Grandilobalide B show a large improvement in fit for ^{13}C chemical shift, which is not reproduced in our results. The results for ^{1}H chemical shifts are reproduced, in the literature a small decrease in the fit to experiment from 0.27ppm RMSE to 0.33ppm RMSE is reported. Both IMPRESSION and our DFT method show a small but significant reduction in fit for the revised structure.

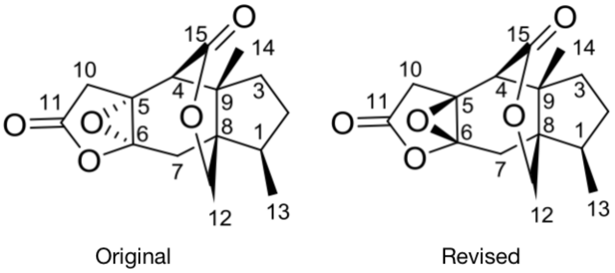

Figure S11 Original and revised structures for Grandilobalide B.

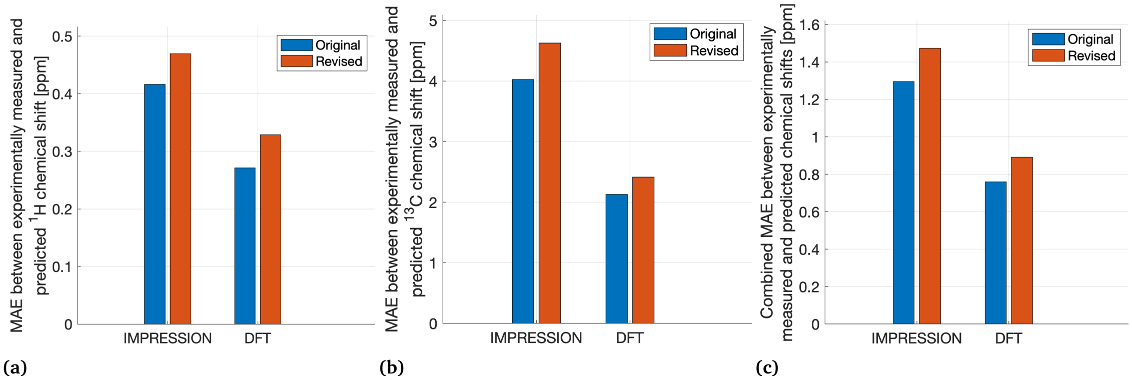

Figure S12 Change in the fit between prediction and experiment for both DFT and IMPRESSION for Grandilobalide B. a) δ^{1}H. b) δ^{13}C. c) Geometric mean across both parameters.

S3.6 Toluene dioxide

In the original work, a large improvement in the fit to experiment for both ^{13}C and ^{1}H chemical shift was reported. The results from our DFT method were inconclusive for both parameters in this case. Pleasingly IMPRESSION mimics the DFT results, irrespective of the DFT methods fit to the reported results.

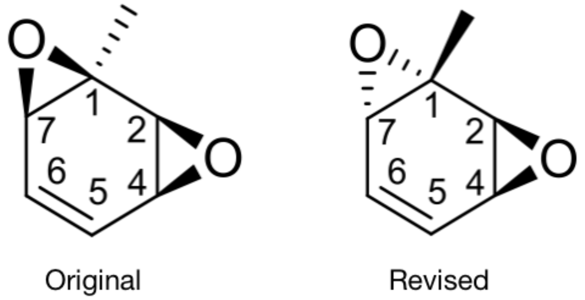

Figure S13 Original and revised structures for Toluene Dioxide

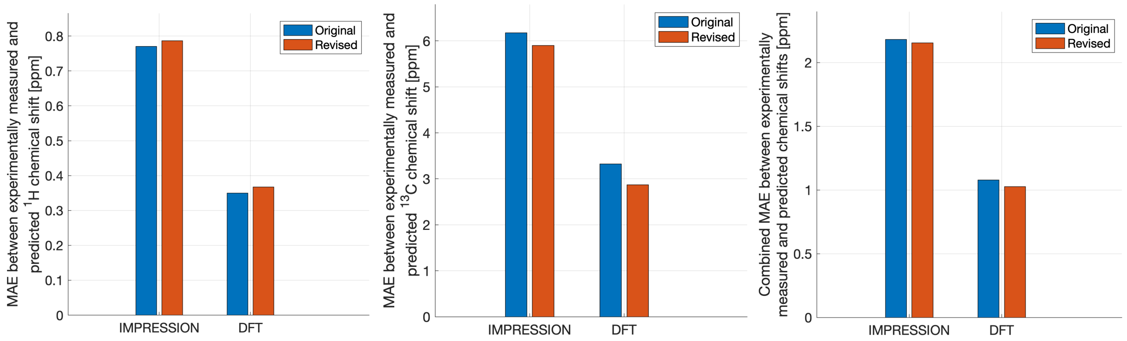

(a) (b) (c)

Figure S14 Change in the fit between prediction and experiment for both DFT and IMPRESSION for Toluene Dioxide. a) δ^{1}H. b) δ^{13}C. c) Geometric mean across both parameters.

S4 Diastereotopic proton assignment in Strychnine

Further analysis was performed to see if the ${}^{1}J_{CH}$ IMPRESSION predictions could be used to assign the diastereotopic protons in strychnine. The ${}^{1}J_{CH}$ values for the 3 sets of diastereotopic protons for diastereomer 1 were compared across the three data sources: IMPRESSION predictions, DFT calculations and experimental measurements.

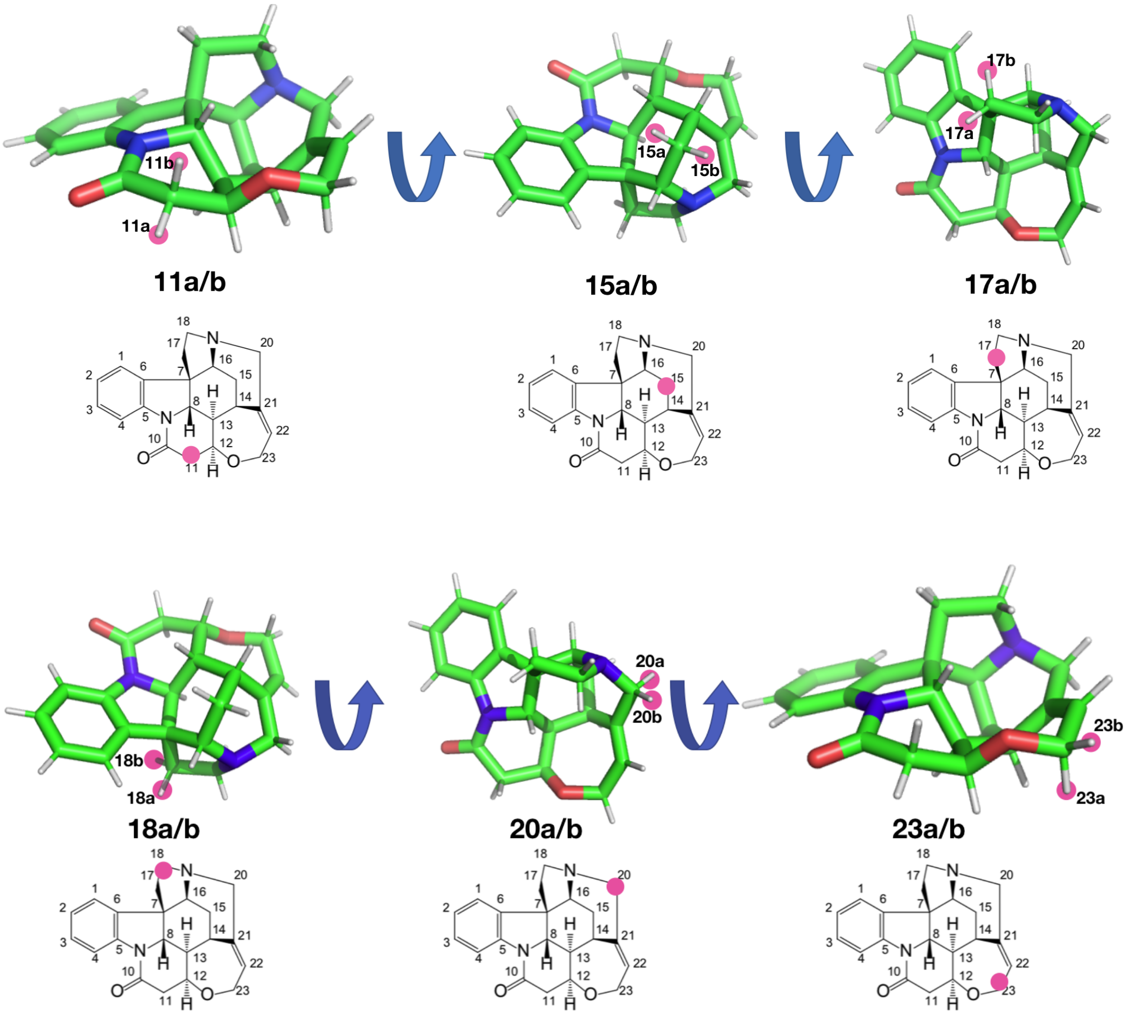

Figure S15 Identification of the diastereotopic protons in Strychnine

The results show that in cases where there is the three methylenes were there is any significant (>2Hz) difference in the experimental ${}^{1}J_{CH}$ values (figures S16a, S16b, S16c) the DFT method and IMPRESSION predictions can distinguish between the diastereotopic protons and correctly assign them. Where the difference in experimental values is small (Figure S17, both DFT and IMPRESSION are not reliable for assignment.

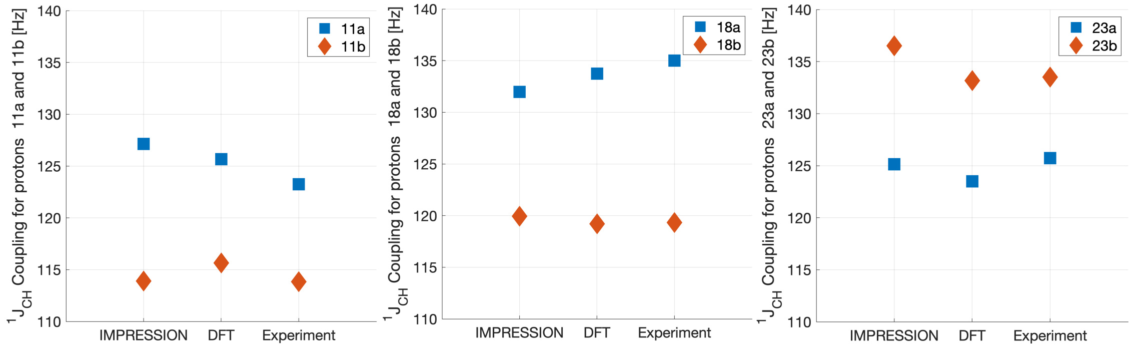

(a) (b) (c)

Figure S16 Comparison of $^1J_{\mathrm{CH}}$ values across all data sources for diastereotopic protons showing significant experimental difference. a) 11a/b. b)18a/b. c)23a/b.

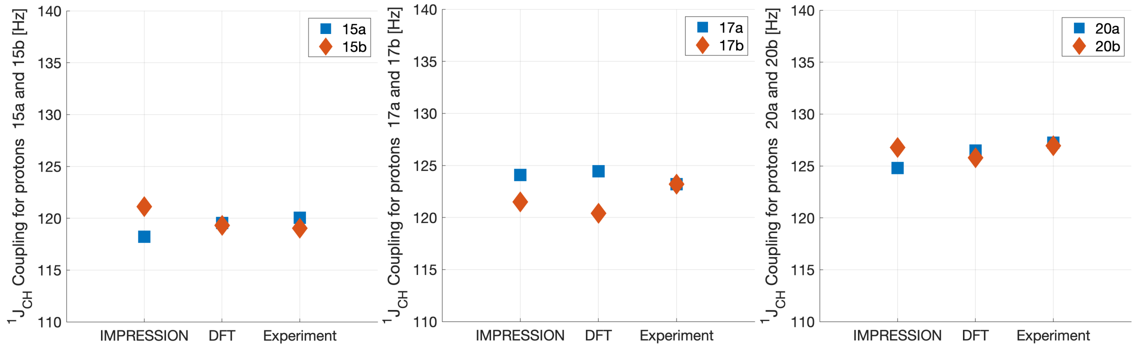

(a) (b) (c)

Figure S17 Comparison of $^1J_{\mathrm{CH}}$ values across all data sources for diastereotopic protons showing small experimental difference. a) 15a/b. b) 17a/b. c) 20a/b.

S5 Strychnine diastereomers

MAE values for each of the parameters across each of the 14 strychnine structures compared to experiment:

Structure	Parameters	MAE [IMP-EXP]	MAE [DFT-EXP]
1a	δ^1H	0.28ppm	0.25ppm
1b	δ^1H	0.73ppm	0.56ppm
2	δ^1H	0.23ppm	0.19ppm
3	δ^1H	0.37ppm	0.35ppm
4	δ^1H	0.69ppm	0.52ppm
5	δ^1H	0.44ppm	0.32ppm
6	δ^1H	0.42ppm	0.35ppm
7	δ^1H	0.77ppm	0.54ppm
8	δ^1H	0.71ppm	0.53ppm
9	δ^1H	0.70ppm	0.66ppm
10	δ^1H	0.48ppm	0.49ppm
11	δ^1H	0.55ppm	0.49ppm
12	δ^1H	0.57ppm	0.36ppm
13	δ^1H	0.73ppm	0.45ppm

Structure	Parameters	MAE [IMP-EXP]	MAE [DFT-EXP]
1a	δ^{13}C	2.26	1.87
1b	δ^{13}C	4.54	3.84
2	δ^{13}C	2.75	2.94
3	δ^{13}C	4.34	3.98
4	δ^{13}C	7.69	7.11
5	δ^{13}C	3.44	2.99
6	δ^{13}C	4.77	3.63
7	δ^{13}C	7.77	8.09
8	δ^{13}C	7.21	7.41
9	δ^{13}C	8.47	7.73
10	δ^{13}C	3.98	3.98
11	δ^{13}C	4.80	4.22
12	δ^{13}C	5.05	3.79
13	δ^{13}C	5.53	3.84

Structure	Parameters	MAE [IMP-EXP]	MAE [DFT-EXP]
1a	${}^{1}J_{CH}$	1.83Hz	1.29Hz
1b	${}^{1}J_{CH}$	4.11Hz	4.06Hz
2	${}^{1}J_{CH}$	2.98Hz	2.22Hz
3	${}^{1}J_{CH}$	3.72Hz	3.64Hz
4	${}^{1}J_{CH}$	5.34Hz	4.94Hz
5	${}^{1}J_{CH}$	3.53Hz	2.98Hz
6	${}^{1}J_{CH}$	2.26Hz	1.83Hz
7	${}^{1}J_{CH}$	6.86Hz	4.45Hz
8	${}^{1}J_{CH}$	5.08Hz	4.73Hz
9	${}^{1}J_{CH}$	8.12Hz	5.24Hz
10	${}^{1}J_{CH}$	3.21Hz	3.04Hz
11	${}^{1}J_{CH}$	2.78Hz	3.26Hz
12	${}^{1}J_{CH}$	4.34Hz	4.26Hz
13	${}^{1}J_{CH}$	3.32Hz	3.58Hz

Structure	Parameters	MAE [IMP-EXP]	MAE [DFT-EXP]
1a	δ^{1}H + δ^{13}C	0.80ppm	0.68ppm
1b	δ^{1}H + δ^{13}C	1.83ppm	1.47ppm
2	δ^{1}H + δ^{13}C	0.80ppm	0.75ppm
3	δ^{1}H + δ^{13}C	1.27ppm	1.18ppm
4	δ^{1}H + δ^{13}C	2.30ppm	1.92ppm
5	δ^{1}H + δ^{13}C	1.24ppm	0.98ppm
6	δ^{1}H + δ^{13}C	1.41ppm	1.13ppm
7	δ^{1}H + δ^{13}C	2.45ppm	2.09ppm
8	δ^{1}H + δ^{13}C	2.27ppm	1.99ppm
9	δ^{1}H + δ^{13}C	2.43ppm	2.25ppm
10	δ^{1}H + δ^{13}C	1.39ppm	1.40ppm
11	δ^{1}H + δ^{13}C	1.62ppm	1.43ppm
12	δ^{1}H + δ^{13}C	1.58ppm	1.17ppm
13	δ^{1}H + δ^{13}C	1.78ppm	1.32ppm

Structure	Parameters	MAE [IMP-EXP]	MAE [DFT-EXP]
1a	$\delta^1H + \delta^{13}C + {}^1J_{CH}$	1.05	0.84
1b	$\delta^1H + \delta^{13}C + {}^1J_{CH}$	2.39	2.06
2	$\delta^1H + \delta^{13}C + {}^1J_{CH}$	1.24	1.08
3	$\delta^1H + \delta^{13}C + {}^1J_{CH}$	1.82	1.72
4	$\delta^1H + \delta^{13}C + {}^1J_{CH}$	3.04	2.63
5	$\delta^1H + \delta^{13}C + {}^1J_{CH}$	1.75	1.42
6	$\delta^1H + \delta^{13}C + {}^1J_{CH}$	1.65	1.33
7	$\delta^1H + \delta^{13}C + {}^1J_{CH}$	3.45	2.69
8	$\delta^1H + \delta^{13}C + {}^1J_{CH}$	2.97	2.65
9	$\delta^1H + \delta^{13}C + {}^1J_{CH}$	3.64	2.98
10	$\delta^1H + \delta^{13}C + {}^1J_{CH}$	1.84	1.81
11	$\delta^1H + \delta^{13}C + {}^1J_{CH}$	1.94	1.89
12	$\delta^1H + \delta^{13}C + {}^1J_{CH}$	2.21	1.80
13	$\delta^1H + \delta^{13}C + {}^1J_{CH}$	2.19	1.84

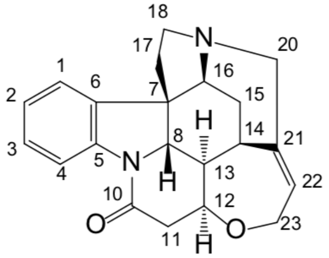

1

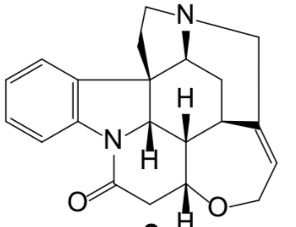

2

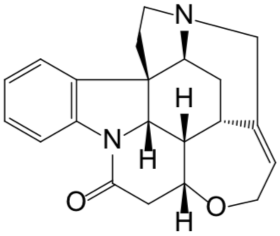

3

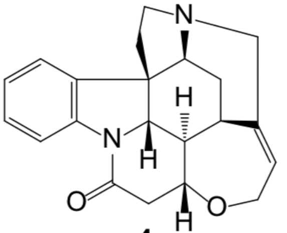

4

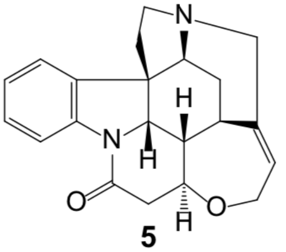

5

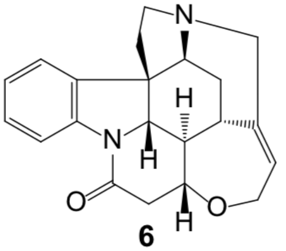

6

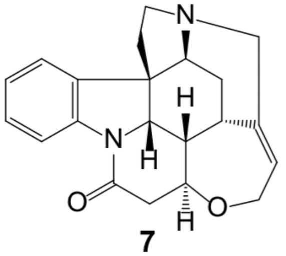

7

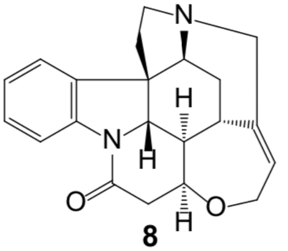

8

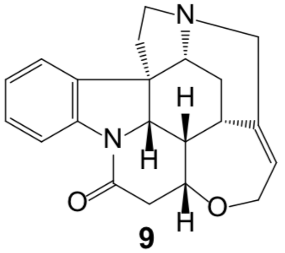

9

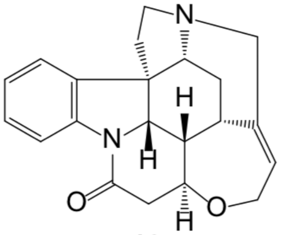

10

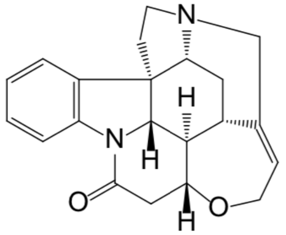

11

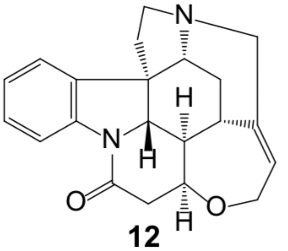

12

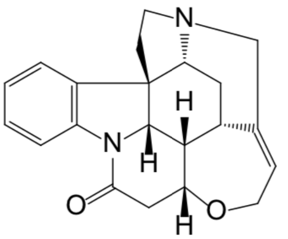

13

Figure S18 The 13 Energetically viable Strychnine diastereomers used for the IMPRESSION validation [22]

S6 Large errors

The largest 5 individual errors between DFT and machine learning for the test set are shown here, for each of the parameters δ^{13}C, δ^{1}H, and $^{1}J_{CH}$.

S6.1 ^{1}H chemical shifts

Mol ID	Atom ID	Error [ppm]	DFT [ppm]	ML [ppm]	Variance [ppm]
YEHWUD	36	11.22	-4.27	6.96	0.63
BEDFUM	6	3.22	3.21	6.42	0.18
IQIKOI	21	2.15	5.34	7.50	0.0023
AROKUN	19	2.01	6.83	8.84	0.0025
WAWQUH	37	1.94	0.76	2.70	0.014

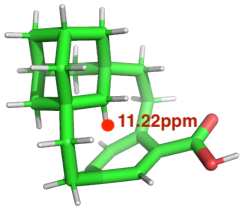

(a) YEHWUD

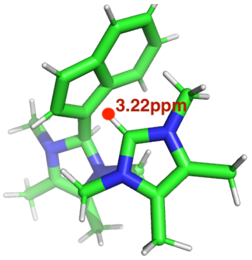

(b) BEDFUM

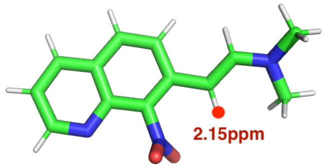

(c) IQIKOI

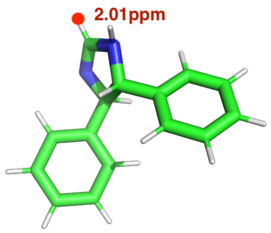

(d) AROKUN

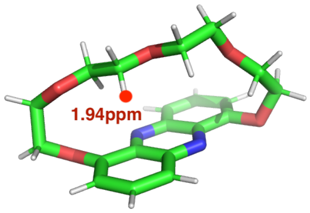

(e) WAWQUH

Figure S19 Biggest errors in ^{1}H prediction.

S6.2 ^{13}C chemical shifts

Mol ID	Atom ID	Error [ppm]	DFT [ppm]	ML [ppm]	Variance [ppm]
DOVWAM	4	-63.33	217.35	154.02	353.15
QUFCEZ	15	42.92	60.82	103.75	45.22
RACGEJ	10	-37.87	180.27	142.40	2.02
BEHWER	5	35.31	115.95	151.26	2.89
QOMVUK	1	32.90	92.98	125.37	8.41

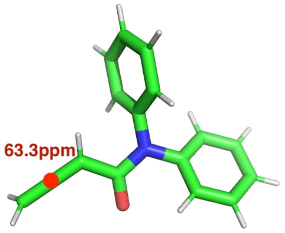

(a) DOVWAM

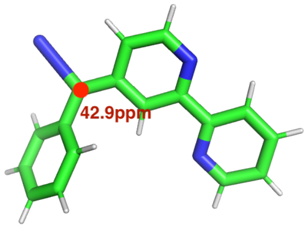

(b) QUFCEZ

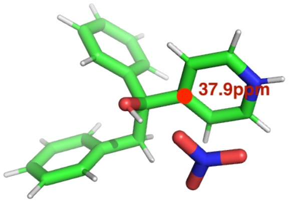

(c) RACGEJ

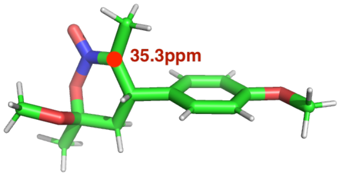

(d) BEHWER

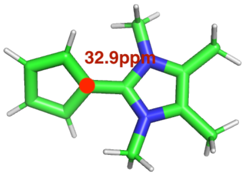

(e) QOMVUK

Figure S20 Biggest errors in ^{13}C prediction.

S6.3 $^{1}J_{CH}$ coupling constant

Mol ID	^{1}H Atom ID	^{13}C Atom ID	Error [Hz]	DFT [Hz]	ML [Hz]	Variance [Hz]
YEHWUD	10	36	24.63	116.31	140.94	365.70
JOTKIM01	50	51	24.40	194.51	218.91	8.52
ZEYLAS	61	70	-18.31	182.64	164.35	3.49
IDURIJ	13	14	-13.37	171.56	158.19	0.13
FEMXOK	7	19	12.13	144.21	156.35	1.01

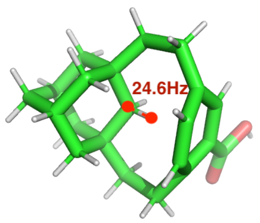

(a) YEHWUD

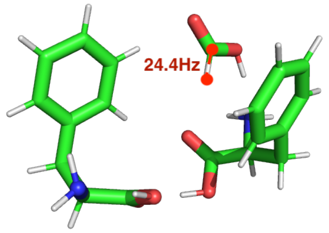

(b) JOTKIM01

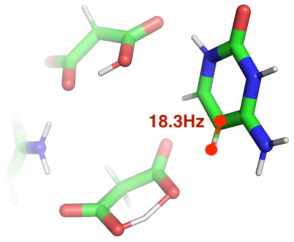

(c) ZEYLAS

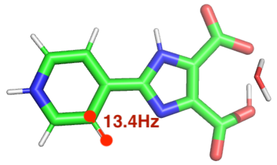

(d) IDURIJ

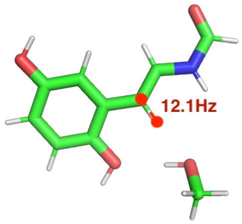

(e) FEMXOK

Figure S21 Biggest errors in $^{1}J_{CH}$ prediction.

S7 Gaussian input files

Example input files for the Gaussian09 software are included here.

```
%Chk=Mol00001_OPT
%NoSave
%mem=26GB
%NProcShared=8
# opt=tight mpw1pw91/6-311g(d,p) integral=ultrafine MaxDisk=50GB

Mol00001 OPT

0 1
 C   -0.03886    1.59169    0.09099
 C   -0.05003    0.06176   -0.01881
 O    0.67385   -0.53818    1.05146
 C   -1.48763   -0.47708   -0.00138
 O   -1.98929   -0.21555    1.30848
 C    0.65858   -0.42520   -1.30698
 O   -0.00173    0.02276   -2.45802
 C    2.11254    0.00797   -1.33359
 O    2.55982    0.61959   -2.27613
 H    0.97884    1.95353    0.26194
 H   -0.42422    2.04115   -0.82812
 H   -0.66421    1.91188    0.92773
 H    0.07032   -0.53935    1.80579
```

Figure S22 Gaussian Optimisation Input File

```
%mem=26GB
NProcShared=8
#T nmr(giao,spinspin,mixed)wb97xd/6-311g(d,p) maxdisk=50GB

Mol00001 NMR

0 1
 C   -0.03886    1.59169    0.09099
 C   -0.05003    0.06176   -0.01881
 O    0.67385   -0.53818    1.05146
 C   -1.48763   -0.47708   -0.00138
 O   -1.98929   -0.21555    1.30848
 C    0.65858   -0.42520   -1.30698
 O   -0.00173    0.02276   -2.45802
 C    2.11254    0.00797   -1.33359
 O    2.55982    0.61959   -2.27613
 H    0.97884    1.95353    0.26194
 H   -0.42422    2.04115   -0.82812
 H   -0.66421    1.91188    0.92773
 H    0.07032   -0.53935    1.80579
```

Figure S23 Gaussian NMR Calculation Input File

S8 CSD structures

Training Data CSD Reference Names

ABIVIQ	BOPKAS	CUDSAX	EFUMUP	FOFQOG
ABOTOC	BOTMUT	CUGLIA	EGAXAL	FOGBIN
ACALIZ	BOVCEW	CUKCAM21	EGOTAW	FOGKIW
ACTOLD05	BOVJOL	CUKSEG	EGUQAY	FOLQUT
ADAZUB	BUBPAQ	CUSFEC	EHAJUS	FOMZUD
ADIDUN	BUCLUI	CUVBIF	EHIYID	FOQNUV
ADOXEZ	BUDHOZ	CUZPAP	EHNPRG	FOTYAP
AFIFAX01	BUGKIX01	CYTOSM13	EKAHOO01	FOVVIV01
AFIGIG	BUGMOG	DAFLIH	ELENEQ	FOWPOW
AFIHOO	BULHIZ	DAJXUI	ELOKIB	FRANAC04
AFUNAR	BULKID	DAQJOV	ELUGOI	FUCVOO
AGAVOU	BUYZUQ01	DAWYEI	EMAQEQ	FUGXIO
AHMVAL	BZCPRO	DEBDIX04	ENIJIV	FULJON
AHUHUH	BZPHAN01	DEBGIB	EREVUS	FULZIV
AHUYUX	CACWAG	DEGREM	ERISII	FUMTOY
AJAPIL01	CAGMIJ	DENPUH	ESTILO03	FUNGAX
AKIGAE	CAHBUL	DETLAQ	ESUQOZ	FUQZEY
AMMCHC11	CANPEM	DETPAU	ESUROZ	FUTWAU
AMUVIP	CASTEV	DEVCIR	ETIROQ	GAMLOV
ANIZUT	CATKAL	DEYTIL01	EVAWEE	GAQLOB
APUREI01	CAXLIX	DIBENZ13	EVAWIJ	GASNEU03
AQUWOY	CBUDCX02	DIBNEH	EVICUJ	GATVED
ARIWAB	CEBKEZ	DICRUD	EVIMUR	GAZPII
ATEZOO	CEBKEZ06	DIFQEP	EVOGOM	GEFLEK
ATOGIB	CEBQIK	DIGGOP	EVOJIK	GEFQIS
ATUJEF	CEFBOH	DIKFEJ	EWODEA01	GEHXEZ
AVALAM	CEGREL	DISJEW	EXOQEO	GELDEI01
AWIZUB	CEHZIY	DIZMOQ	EYIKUS	GENFUA
AWOTAH	CEKPIR	DLALNI14	EYOGEG	GEYTIN
AXADAF	CEKYAS	DLHTDA10	EZUJIU	GICCEA
AXADUZ	CELRAP	DLTYRS	EZUTIC	GILKIW01
AXEHAO01	CEMBED	DMTCUN10	FABVUC	GIMGIU
AXMQOL	CEPKIS	DMXNPY	FACQUV	GITNEE
AYEROL	CIBFEA	DNPHOL	FACWUC	GIVHOJ
AZIWUD	CIFSIV	DODWOI	FAFXUF	GOCCOS
BAFDIV	CIGJUX	DOFGEK	FAHPAH	GODSOH
BAJYOB	CIKBUU	DOKVUV01	FAMFII	GOJVUY
BANJOQ	CIPBAF	DOPSAC	FASZOP	GUFXOV
BAPQOA	CIQHOA	DOQDET	FATBEI	GUHXOY01
BAPYAU	CIQYAD	DOSZES	FAVYIN	GUKXIT
BASDOO	CIRGOB	DOTPOS	FAZRED	GULDIA
BASHUA	CISXOT	DOVGUR	FECQAF	GUMMOZ01
BATVEY	CITQAY	DOYVUK	FEFYEX	GUYBOR01
BAVZEE	CIXGOF	DUCWAA	FEGFIG	HAFDIC
BAYZUW	CMXMCH	DUDDOV	FEHLEL	HAHVIY
BEDJOM	COCPAN	DUDKUJ	FEKDUU	HAKWUN
BEFJAY	COFGUA	DUFVEG	FEQFIT	HALNEP
BEJTEP	COFNUI	DULJEA	FERTON	HALVAT
BELHAB01	COGMOB	DUNLAA	FESNOG	HAMDOP
BEMZAV	COLYIN	DUNSAH	FESQAX	HATXIJ
BEXNUO	COMXOR	DUNTOV	FEVHEV	HAWTEF
BEZREF	CONNUP	DUSJAD	FEWSEH	HDPDXZ
BIBXIT02	COTMEE	DUSWIY	FICLEK	HELYOM01
BICVIS01	COWLUX	DUTTAN10	FICTOC	HEQWOQ
BIFFAZ	COXXIY	EBIWEU	FIHNUH01	HEVDIW
BIWZOX	COYREO	ECASAC	FIJQAQ	HEXVAI
BOAYPI	COYSIS	ECIPIR	FIKCAE	HIFGEJ
BOCHIL	CTOGBS20	ECMPCA	FIYBEU	HIFPIX
BOGFUA	CTPROL10	EDEKOQ	FNPEYO	HIFQET
BOMBEK	CUDDUB	EFIKOT01	FOCBEF	HIGCIK

Training Data CSD Reference Names

HIYHAY
HMCNSP
HNOBCH
HOCPUL
HOMCOD
HOPKUT
HOQSIQ
HOVFUT
HOWWOH
HOZBII
HOZGAG
HURLAI
HXMTAM10
HXOCTM
IBUYIQ
ICAPOR07
ICEMIO01
ICOYEE
IDILUD01
IGENOZ
IHANAG
IHOQUT
IJIHOA
ILAJIQ
ILIMEV02
IMUXOF
INACET03
IQIDIV
IQIZAK
IQOROW
IQUFUX01
ITAFEP
ITIKEB
ITUVOI
IVAKAS
IVEREH
IVIDAS
IVIHAY
IXOYEA
IYASUW
JABKUV
JAPBIO
JAWCIW
JAXHEW
JECNUD
JEDTIV
JEGTUN
JEXBOE
JINHET
JOCDAG
JONQOU
JOTBAV
JOYGEJ
JOZYUU
JUMCEB
JUNJIN
JUPJAH
JUSQUL
KABHED
KACNIN
KADDIE
KAGZIE
KAMROH
KATKIA
KAVCOC
KAYHIE
KEDRER
KEMHAL
KESTAD
KIBKAJ
KIGQIA
KIHXUW
KIMSUU01
KINGUJ
KIXROA
KIZVEV
KOCKET01
KOKLIH
KONTIQ01
KOPBAS
KOTJAE
KOVFUW
KOWCAC
KOXBEE
KUGKAZ
KUKCUP
KUQFUY
KUVBEI
KUVKES
KUVWON01
KUWZOS
KUXJIY
KUYNOH
LACVAM
LAFHEH
LAVCET
LEGXUS
LEHJAM
LEMVEH
LEPPIF
LERJAV
LESCET
LEZJUV
LGLUAC13
LIHMOG
LILDEP
LILJOG
LIWFEC
LIYPEO
LOCVEE
LOKDEW
LOMHOK
LOMNUY
LOSMOW
LOVCAC
LUPGAG
LUQSOG
LUQYIG
LURVUR
LUVPEX
MAMKAO
MAPLIZ01
MAQWIM16
MATGOG
MATPEC
MATVAE
MAXDUL
MECZID
MEDLEN
MEGNES
MEHPIB
MELVAA
MENNAV
MENSEE
MEQFAS
MESYIS
METAMI02
MEWROX
MEYCIC
MEYTUH
MEYWOC
MEZHEG
MIDXIH
MIHZUZ
MIMREG
MIMTAE
MINGAR
MIPYAL
MIQNEF
MIVTUG
MIWQIS
MIXWEX
MNPYDO10
MOBXAC
MOFCOA
MOGYIR
MOLQUB
MOYKUG
MTHPRG
MTYROS01
MUGDID
MUHZUM
MUKBUR
MULBIE
MUNWUP
MUVCAI
MVAHIV
NACGOP
NADVIX
NAFHOR
NAMZAC
NAMZEG
NAPHTA23
NAPTYR11
NASRUV
NATNAA
NAXRUC
NAYPAF
NAYZOD
NEDYEA
NEFHOY
NEMZAG
NEPXIR06
NEPXOX
NESZOB
NETIND01
NEWREN
NEXMOT
NIFBEJ
NIFJOB
NIFRAX
NIHNEY
NIJKEX
NINWEO
NIPYAZ
NISMAD
NIVJAE
NIVMIQ
NIYWID
NOFYEM
NOQBUQ
NOVDOR
NUBLOL
NUHFEB
NUKJIO
NUKXEX
NUPQEU
NUQHIR
NUYWIP
OBOWOU
OCEHIP01
OCOPOL
OGOXEP
OHIWUX
OJAQOH
OLOJAB
OLOREM
OMCHDO
OMOMOS
ONILAZ
OPOZAW
OQUHEP
ORIDAW
OTAKEB01
OWOHAL01
OXOFMB
OZICAC
PABBIF
PADTIX
PADXOJ
PAFGUA
PAGLEO
PAGWIG
PAJDOU
PAJVOO
PARHAR
PAXCEX
PAYJEH
PEFSID
PEGLUL
PENBUH
PENTYN
PEPGEW
PEXFUT
PEXLAH
PEZFEG01
PHTHAC02
PHTHAC06
PIBGOX
PIGROM01
PIGTAC
PINVOX
PINYIW
PIPINE01
PIPINE11
PITQIS01
POBDER
POBSAB
POQVUO
POQWOJ
PORROE
POSJAI
POVJAL
POZWUW
PUDDUP
PUQNUK
PUQTAW
PUYTAE
QACVAT
QAHSOI
QAJBUZ
QAKJUJ
QAKMOG
QALZUA
QANQUR
QAPJIA
QAPNAZ
QAPVOT
QATVIS
QAZMIP
QEBBUW
QECHEO
QEPNUW
QEYRER
QIKJIF
QIMKIG03
QIQYIA
QIRLUA
QIWGEJ
QIWMUG
QOVREZ01
QUDREM
QUVPOO
QUWJOJ
QUYJUQ
RAFINO01
RALQUR
RAMZEL
RAYXEU
RAYXOH

Training Data CSD Reference Names

REBXON
REDYAB
REGFER
REGKIX01
REGYEJ
RELCUH
REYCII
REZJUC
RIFBUE
RIGVEJ
RIQWIZ
RIWNEQ
RIXXOM
RIZWUS
ROLVEV
ROSLAO
RUCFAX
RUGCED
RUGQOA
RUJQOE
RUJSAS
RURRAY
RUVSAC
RUWJAU
RUWMAX
RUWQIK
RUZXIU
SADJEM
SADXOL
SAGQUO
SAHCOV
SAHZAF
SAKJUM
SANWEJ
SAPHAU
SARJED
SAWHUV
SAWJUX
SAYTAN
SAYWOG
SAZLAH
SECTIF
SEDMOD
SEHNAW01
SEJWOT
SELKEB
SEQREN
SIQQEP
SITCUU
SIVJOY
SIWDEH
SIYYUU
SOPLEO
SOXHAQ
SUCACB12
SUCANH12
SUCROS47
SUCTAN
SUFGAB
SUHYIE
SUPKET
SUSYAI01
SUVCUJ
SUXCAQ
SUXROS
SUZJAZ
TABBOQ
TABNIV
TACRIB02
TAHMOE
TALHAR
TALNAV01
TAMLID
TANBEP
TANTEK
TAPCIW
TARGEB
TARGUO
TATNEI
TECQEX
TEGVUW
TEJREG
TEKSOR
TELKAZ
TENMIK
TEPHME02
TEVLIQ
TICBUD
TIHBAO
TIMHED
TIQNIQ
TIQWOG
TIXPOF
TMXSTQ10
TOHVIW
TOPROG
TOPSEW
TOVSUS02
TPHETY01
TUCJEI
TUCNUC
TUJJEP
TULDAH
TUNCOW
TUNTUT
TUSQUU
TUWCEU
UBEBAG
UBUPEM
UCOMOO
UCOQAE
UCUZOJ
UDEHER
UFAGOY
UHADOX
UKUTUP
UPACUK
UPADOG
UQIMUE
URAHIF
URAWEQ
URESOB
USUZUF
UTAGAZ
UTEJIO
UTIHOV
UVIMES
UWACEB
UXICAH
UYIREB
UYUDUO
UZUHED
VACLAM02
VAJVOU
VAPCEW
VAWJAG
VAXLAJ
VEBWEH
VECSAZ
VEFPIF
VESHUX
VEXCUW
VEZNOF
VIBZUB
VIDFEV
VIGWOY
VIGXAK
VIHBIZ
VOFSEP
VOKXOJ
VOLKIS
VUDKIP
VUFGEI01
VUFSEU
VUFWAV
VUKFOY
VUNFUF
VUPHIZ
VUTBUI
VUTNAB
VUZQOX
WABTAU
WACZUX
WADGEO01
WADQID
WAGBEO
WALNEC
WANVEP
WAQNUZ01
WAZMAL
WECXUZ
WESVIZ
WEWTUP
WIBWIN
WIBXUA
WIFZOC
WIPHAG
WIVYUV
WIYDUF
WIZZAI
WOBLAA
WOBWUF01
WOGQEO
WOJGUX
WOJHAG
WOKPER05
WOLNIW
WOZPUW
WUCJOV
WUKLAP
WUSQUY
WUWMEG
WUYMUZ
XAKLUR
XAVMUE
XAVZOJ
XAXHOW
XAYDIK
XAZQOF
XAZROH
XEBYUA
XEDNAX
XEDTEG
XEHTUZ
XEMDAX
XENLAE
XETMAL
XEVCEH
XEWNES
XEXQOH01
XEYRIE
XEZYIK
XIJFEB
XIMCOL
XIMJAE
XINJIN
XISHOY
XIVVAA
XIWREA02
XOBGAY
XOGWAR
XOGXEX
XOMJIS
XOWDAQ
XUHPIB
XUPYIR
XUVSUE
XUYZIC
YAGJEX
YAMHID01
YAPBUO
YAPZEU
YAQWAR
YARDUQ
YAWWAU01
YAYDIN
YEJPAG
YEJZES
YEKVEQ
YENLAF
YEXZIM01
YIDPEG
YIDPIM
YIFWAM
YIGSUE
YIHHON16
YILYOJ
YOGSIY
YOKYOO
YONBOT
YOPLIY10
YOWRAF
YOXGIB
YUCQUJ
YUDLAM
YUDMOZ
YUDPAQ
YUFYED
YUHTEA03
YUHTOK
YUNTOR
YUNYIR
YUQCUJ
YUQMED
ZAJHOH
ZAJVAK
ZETHUD
ZEWPUM
ZIFKEG
ZILQOA01
ZIYSIL
ZODXEV
ZOFCUU
ZOLBUX
ZONYUY
ZOZTOX
ZUPGIA10
ZUPGUM
ZUPHAT
ZUQVOY
ZZZLUK05
ZZZMBS02

Testing Data CSD Reference Names

ACRDIN07	DAFTAF	GADSIO	KETYUF	OGIMIC
AFIQUC	DAJZEU	GADVAJ	KOFKAR	OHEWOP
AHATEK	DASNIV	GAQJUF	KOGWUZ	OJICUF
AHOWOL	DENXUP02	GASXON	KOJTOT	OMABEK
AHOXOL	DILDUZ	GAWFEQ	KOTMUB	OMSTER01
AJIXUM	DILKIT	GIDHUW	KUJZIY	ONBZAM
AKUBIT	DITZOX	GIXKOP	KUTKAL	OPIZAQ
ALEXEW	DIWWEN	GIZFEB	KUYWEH	OWIWUN
ALOSEZ	DIZWEQ	GIZRUE	KUZJIA	OXAROV
AMEXOH	DOHPEV	GOVQOX	KUZQIG	OXUJUN
AMUQOQ	DOLBIR10	GUCJUK	LADNEL	PACWAU
ANAHII	DOMNEY	GUFYOX	LAFHEH	PANLEZ10
ANOSAY	DORKOK	GUJGEX	LAVSIL	PEDHAJ
APODUG	DOTFOI	GUTZOM	LEVSIO	PEFGIS
APUPIK	DOVWAM	HABNED	LILDEP	PELXAG10
AQAGII	DUTKOU	HAMTIZ	LIXQEO	PEPLAX
AQEYAW	DUZLUF	HAXREE	LIZHEJ	PETRAH
AROKUN	DXCYTD	HECNOS	LOPLUZ	PEWNIQ
ARONOM	EABZBU	HESTOO	LUDZIT	PEXPEN
ARUZUK	EBAXOW	HIMSUS	LUQDOS	PHBZAC01
ASPARM10	EBOVEX	HISNII	LUXSAY	PIHBOZ
AWAVEZ	ECODUV	HIWYIV	MAHPUJ	PIJREF
AXADAF	EDAXOW	HIZHOP	MALSOH	PILFIB
AXAWIG	EDIZUM	HODKEQ	MAQWIM23	POHCAS
AXOSOW03	EFIBAX	HODLOC	MATQOO	POKKAD10
AYUNEO	EHAHAY	HOMKIF	MEHLER	POLJEF
AZIDES	EKAHOP	HOMZUG	MEHNAP	PRMDIN05
BAJCIY03	EKAWAQ	HONKEC	MEJDOU	PUMQEV
BAPPUF	EKOGAO	HUDHEU	MEJQEY	PUNFAH
BAQNEM	ELAWIX	HUDYUA	MELAMI05	PUPBAD01
BASNOZ	EMEFOT	HUVWOL	MENDAL01	PUWNIG
BAWRAT	EMIPUM	HUYYOP	MESQOR	PYAZAC
BAYPAT	EMISUQ	IBEHII	MEYBIB	QAKDAJ
BEDFUM	EMODUG	IBOPIA	MISDAT	QAMKEW
BEDLEB01	ENIMET	IDUJEW	MOBNUM	QECNAP
BEGDIB01	EPHEDR01	IDURIJ	MODXUZ	QEPRIO
BEHWER	ESESEA	IJEZUS	MOSLAI	QEXKUA
BERSOG	EVIHUM02	INAVIC	MOTNUF	QIYLAM
BIKNUE	EVILEB	IPINIE	MUBBAN	QOMVUK
BIXQEF	EVINII	IQIKOI	MUJGEE	QQQAMS02
BOLGOZ	EVIQEF	IQIZEO	MUTWON	QUFCEZ
BOMSIH	EWOBIB	IQUBZA	NAJLUF	QUFJUY
BOPJAS	EXEWEJ	IQULUC	NANJIW	QUWFIZ
BUFNEV01	EXEYUD	IROZIY	NAPTPR	RACGEJ
BUGQUQ	EXUVUP	ISIJIE	NASZAJ	RAKTOO
BUMNOM	EYASAZ	ITINEG	NBZOAC11	RAVFOK
BUZJIR	EZISUC	ITIREI	NCUBEB10	RECYIH
BZAMID08	FACZIU	IVABEO	NEQPEG	REKMEZ
BZTROP11	FADHOJ	IVEZAK	NEVDOH	RICTIG
CAZCOX	FAHLAB	IYASUW	NEZFON	RIHFIY
CBMZPN21	FAHXUH	IZAKOK	NIQTAJ	RIMHEC
CIKSAQ	FAJDEC	JESHIZ	NORFUW	RIZBAF
CINCHO10	FELDOR	JIPCUG10	NUKSAO	ROGRIQ
COCYAW	FEMGAF	JOQTUE	NUQLES	ROHJED
COLBAG	FEMXOK	JOTKIM01	NURZOP	ROJHOP
CORTPY	FEPTID	JULGOO	OCATOC	ROJXOD
COWPUZ	FEZLUT	KABKIJ	OCAWOF	RUCNOU
COYBOJ	FIHLEO	KAHJEK	OCIPAR	RUKTAU
CUTCUQ	FOSLEG	KAKHEL	ODOROO	RULDAF
CXMTUN	FUPWES	KEMFIS	OFEVOL	RULHOX

Testing Data CSD Reference Names

RUVPIJ
SAJCAJ
SATPEI02
SATPUZ
SAVREN
SAWVET
SAZFOO
SEBVAW
SEFNOG
SENKUR
SEYCUU
SIGSAD
SIHCES
SIHZAM
SOGCUN
SORFIQ
SUHFEH
SUKNIW02
SUWKEC
SUYYIV
TAJSOM
TAVJAD
TEMKAZ
TESDOL
THYDIN05
TICLIC
TIWZUV
TOPRIB
TOPXUT
UBUXOG
UCANIV
UJUKIT
UKUROJ
UMUKUJ
UNAMOL
UNURIF
UNUVEF
UQAMIK
UQOLIW
UWEZED
UWOCAM
VAFPAV
VANFEV
VASLOR
VEQMUA
VETJIO
VEZCUY
VIDDAO
VIDMAX02
VILPUB
VOCHUR
VOGDIE
VONNOB
VOXNOL
VUDDUV
VUHZEE
WAFBIQ
WAWQUH
WECZEJ
WEVVEZ
WIFQEI
WIHBEW
WIQZOL
WOBRIP
WOKJOV
WUCVIB
XABFUE
XAQTUF
XASHUW
XAZYIG
XEZFUF
XIMGAB
XINHIL
XIYTIJ
XIZVAD
XOFFEF
XOHMAI
XOWJUP
XUJKUK
XULNOI
XUVBAT
YAZDEI
YEGGIA
YEHWUD
YERTIZ01
YIDTIQ
YIMPOB
YIPPOC
YIXPUR
YOCWUK
YODPAJ
YOFTOE
YOWYOY02
YOXRIO
YUNYUC
ZATDOP
ZAYPOE
ZEBXOV
ZEMNAG
ZEYLAS
ZIGBAS
ZIKQIT
ZIWMOJ
ZIYYUD
ZOFNUD
ZOSVEI
ZOXYOA
ZOYMOP07
ZZZBPY10
ZZZFFY01

S9 Full Gaussian reference

Gaussian 09, Revision D.01, M. J. Frisch, G. W. Trucks, H. B. Schlegel, G. E. Scuseria, M. A. Robb, J. R. Cheeseman, G. Scalmani, V. Barone, G. A. Petersson, H. Nakatsuji, X. Li, M. Caricato, A. Marenich, J. Bloino, B. G. Janesko, R. Gomperts, B. Mennucci, H. P. Hratchian, J. V. Ortiz, A. F. Izmaylov, J. L. Sonnenberg, D. Williams-Young, F. Ding, F. Lipparini, F. Egidi, J. Goings, B. Peng, A. Petrone, T. Henderson, D. Ranasinghe, V. G. Zakrzewski, J. Gao, N. Rega, G. Zheng, W. Liang, M. Hada, M. Ehara, K. Toyota, R. Fukuda, J. Hasegawa, M. Ishida, T. Nakajima, Y. Honda, O. Kitao, H. Nakai, T. Vreven, K. Throssell, J. A. Montgomery, Jr., J. E. Peralta, F. Ogliaro, M. Bearpark, J. J. Heyd, E. Brothers, K. N. Kudin, V. N. Staroverov, T. Keith, R. Kobayashi, J. Normand, K. Raghavachari, A. Rendell, J. C. Burant, S. S. Iyengar, J. Tomasi, M. Cossi, J. M. Millam, M. Klene, C. Adamo, R. Cammi, J. W. Ochterski, R. L. Martin, K. Morokuma, O. Farkas, J. B. Foresman, and D. J. Fox, Gaussian, Inc., Wallingford CT, 2016.

Notes and references

[1] C. Saunders, A. Gammerman and V. Vovk, 1998.

[2] M. Rupp, R. Ramakrishnan and O. A. Von Lilienfeld, *J. Phys. Chem. Lett.*, 2015, **6**, 3309–3313.

[3] B. Huang and O. A. von Lilienfeld, *arXiv preprint arXiv:1707.04146*, 2017.

[4] F. A. Faber, A. S. Christensen, B. Huang and O. A. von Lilienfeld, *J. Chem. Phys.*, 2018, **148**, 241717.

[5] A. S. Christensen, L. A. Bratholm, S. Amabilino, J. C. Kromann, F. A. Faber, B. Huang, A. Tkatchenko, K. R. MÃijller and O. A. von Lilienfeld, *QML: A Python Toolkit for Quantum Machine Learning*, 2019, `https://github.com/qmlcode/qml`.

[6] H. S. Seung, M. Opper and H. Sompolinsky, Proc. 5th Ann. Work. Comp. Learn. Theory, New York, NY, USA, 1992, pp. 287–294.

[7] M. Gastegger, J. Behler and P. Marquetand, *Chem. Sci.*, 2017, **8**, 6924–6935.

[8] J. S. Smith, B. Nebgen, N. Lubbers, O. Isayev and A. E. Roitberg, *J. Chem. Phys.*, 2018, **148**, 241733.

[9] F. M. Paruzzo, A. Hofstetter, F. Musil, S. De, M. Ceriotti and L. Emsley, *Nat. Commun.*, 2018, **9**, 4501.

[10] M. Frisch, G. Trucks, H. Schlegel, G. Scuseria, M. Robb, J. Cheeseman, G. Scalmani, V. Barone, B. Mennucci, G. Petersson and s. S. S. o. S. I. others (for the full reference, *Wallingford, CT*, 2016.

[11] C. Adamo and V. Barone, *J. Chem. Phys.*, 1998, **108**, 664–675.

[12] A. McLean and G. Chandler, *J. Chem. Phys.*, 1980, **72**, 5639–5648.

[13] R. Krishnan, J. S. Binkley, R. Seeger and J. A. Pople, *J. Chem. Phys.*, 1980, **72**, 650–654.

[14] P. B. Wilson, M. Grootveld and S. C. L. Kamerlin, *Magn. Reson. Chem.*, 2019.

[15] J.-D. Chai and M. Head-Gordon, *J. Chem. Phys.*, 2008, **128**, 084106.

[16] W. Deng, J. R. Cheeseman and M. J. Frisch, *J. Chem. Theory Comput.*, 2006, **2**, 1028–1037.

[17] M. W. Lodewyk, M. R. Siebert and D. J. Tantillo, *Chem. Rev.*, 2011, **112**, 1839–1862.

[18] R. Laskowski, P. Blaha and F. Tran, *CHESHIRE Chemical Shift Repository*, 2019 (accessed October 2nd, 2019).

[19] F. Nogueira, *A Python implementation of global optimization with gaussian processes*, 2019, `https://github.com/fmfn/BayesianOptimization`.

[20] T. A. Halgren, *J. Comput. Chem.*, 1996, **17**, 490–519.

[21] A. G. Kutateladze, D. M. Kuznetsov, A. A. Beloglazkina and T. Holt, *J. Org. Chem.*, 2018, **83**, 8341–8352.

[22] A. V. Buevich, J. Saurí, T. Parella, N. De Tommasi, G. Bifulco, R. T. Williamson and G. E. Martin, *Chem. Commun.*, 2019, **55**, 5781–5784.
